# Interleukin-6 Modulates the Expression and Function of HCN Channels: A Link Between Inflammation and Atrial Electrogenesis

**DOI:** 10.3390/ijms252212212

**Published:** 2024-11-14

**Authors:** Valentina Spinelli, Annunziatina Laurino, Valentina Balducci, Manuela Gencarelli, Jessica Ruzzolini, Chiara Nediani, Giulia Elena Mandoli, Matteo Cameli, Leonardo Sacconi, Laura Sartiani, Elisabetta Cerbai

**Affiliations:** 1 Department of Neuroscience, Innovative Treatment, Drug Research and Child Health, University of Firenze, 50139 Firenze, Italy; valentina.spinelli@unifi.it (V.S.); annunziatina.laurino@unisi.it (A.L.); valentina.balducci@unifi.it (V.B.); manuela.gencarelli@unifi.it (M.G.); jessica.ruzzolini@unifi.it (J.R.); 2 Department of Molecular and Developmental Medicine, University of Siena, 53100 Siena, Italy; 3 Leon H. Charney Division of Cardiology, New York University Grossman School of Medicine, New York, NY 10024, USA; 4 Department of Experimental and Clinical Biomedical Sciences, University of Firenze, 50139 Firenze, Italy; chiara.nediani@unifi.it; 5 Department of Medical Biotechnologies, Division of Cardiology, University of Siena, 53100 Siena, Italy; giulia.mandoli@unisi.it (G.E.M.); matteo.cameli@unisi.it (M.C.); 6 Institute of Clinical Physiology, National Research Council, 50139 Florence, Italy; leonardo.sacconi@cnr.it; 7 Institute for Experimental Cardiovascular Medicine, University Heart Center and Medical Faculty, 79110 Freiburg, Germany; 8 European Laboratory for Non-Linear Spectroscopy-LENS, Sesto Fiorentino, 50019 Firenze, Italy

**Keywords:** IL6, HCN channels, inflammation, atrial electrogenesis, cardiomyocytes

## Abstract

Inflammatory cytokines, including interleukin 6 (IL6), are associated with ion channel remodeling and enhance the propensity to alterations in cardiac rhythm generation and propagation, in which the hyperpolarization-activated cyclic nucleotide-gated (HCN) channels play a crucial role. Hence, we investigated the consequences of exposure to IL6 on HCN channels in cell models and human atrial biopsies. In murine atrial HL1 cells and in cardiomyocytes derived from human induced pluripotent stem cells (hiPS-CMs), IL6 elicited STAT3 phosphorylation, a receptor-mediated downstream signaling. Downregulation of HCN1,2,4 by IL6 was observed after 24–48 h; in hiPS-CMs, this effect was reverted by 24 h of application of tocilizumab, a human IL6 receptor antagonist. In parallel, hiPS-CM action potentials (APs) showed a reduced spontaneous frequency. Moreover, we assessed IL6 and HCN expression in dilated left atrial samples from patients with mitral valve disease, an AF-prone condition. IL6 levels were increased in dilated atria compared to controls and positively correlated with echocardiographic atrial dimensions. Interestingly, the highest IL6 transcript levels and the lowest HCN4 and HCN2 expression were in these samples. In conclusion, our data uncovered a novel link between IL6 and cardiac HCN channels, potentially contributing to atrial electrical disturbances and a higher risk of dysrhythmias in conditions with elevated IL6 levels.

## 1. Introduction

The hyperpolarization-activated cyclic nucleotide-gated (HCN) channels, a family of non-selective channels generating the so-called *f*-current, play a major role in cell rhythmicity, including sinus node excitability and atrioventricular conduction [1]. At the neuronal level, transcriptional and post-translational modulation of HCN channels by inflammatory mediators has been associated with degenerative disorders and neuropathic pain [2,3,4]. 

Cytokines from the interleukin-6 (IL6) family have emerged as key players and potential predictors of cardiovascular disease onset and prognosis. The recent results obtained in clinical trials aiming to target inflammation support the role of IL6 in this context: in the CANTOS (Canakinumab Anti-Inflammatory Thrombosis Outcomes Study), only patients with on-treatment decrease in IL6 levels after the first dose exhibited a significant reduction in the incidence of major cardiovascular events [5,6]. 

A likely association between arrhythmic events and cytokine bursts, including IL6, was suggested in patients with autoimmune diseases and, more recently, in subjects hospitalized for severe COVID-19 [7]. In vitro studies from the same group showed that molecular alterations occur in the expression of potassium channels and connexins, possibly related to cardiomyocyte electrophysiological abnormalities [8,9]. Yet given the major role of *f*-current in nodal cardiomyocytes, the frequent occurrence of bradycardia or atrioventricular conduction block [10] are suggestive of an effect on HCN channels and/or their regulatory β-subunit MiRP-1. Indeed, a link between the increased cytokine galectin-3 and the downregulation of sinus node HCN was recently shown in a murine model of heart failure [11]. 

Based on these observations, we hypothesized that HCN channels, constitutively present in several cardiac cell types, and their regulatory β-subunit MiRP-1 may represent key targets of IL6. First, we combined different in vitro cardiac cell models to gain insight into the molecular and functional hallmarks of the interplay between IL6 and HCN channels. Hence, we chose atrial pathological dilation as a relevant clinical setting predisposing to electrophysiological remodeling. Through the analysis of left atrial samples from patients undergoing cardiac corrective surgery, we verified the association between enhanced expression of IL6 in the tissue and downregulation of atrial HCN expression.

## 2. Results

### 2.1. IL6 Signaling Hampers HCN Expression in HL1 CMs

**Figure 1:**
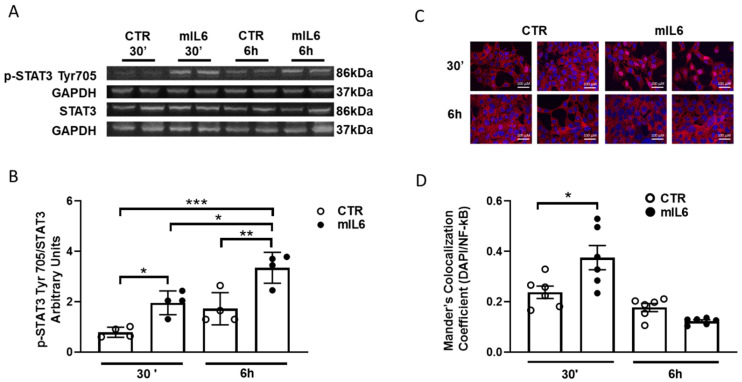
IL6 induces STAT-3 phosphorylation and nuclear staining of NF-kB in HL1 cells. HL1 cells were grown in normal medium supplemented or not with mIL6 (50 ng/mL). (**A**,**B**): Western blot analyses of HL1 cells treated with mIL6 for 30 min and 6 h. Protein levels of total and phosphorylated STAT3(Tyr705) were determined by Western blotting. GAPDH was used as an endogenous control. (**A**): Representative immunoblots from each experimental group. (**B**): Densitometric analysis showing data represented as the mean ± standard error of four independent experiments. (**C**): Immunofluorescence labeling of NF-kB (red) after 30 min and 6 h treatment with or without IL6. Nuclei are stained in blue (DAPI). (**D**): Mander’s colocalization coefficient indicating the level of signal colocalization between DAPI and NF-kB. Data are represented as the mean ± standard error of six independent experiments * *p* < 0.05, ** *p* < 0.01, *** *p* < 0.001, *p* > 0.05 for CTR vs. mIL6 after 6 h. Ordinary one-way-ANOVA.

To validate our experimental conditions, we first assessed whether exposure to murine IL6 (mIL6) was able to promote an intracellular cascade in HL1 cells, a murine atrial cell line. Figure 1A,B show a significantly increased phosphorylation of STAT3 from 30 min up to 6 h after exposure to 50 ng/mL mIL6. In keeping with the activation of a rapid intracellular cascade by mIL6, 30 min exposure induced a significant increase in nuclear translocation of NF-kB, which returned to values similar to control after 6 h (Figure 1C,D). Within this time interval, mIL6 did not alter cell viability (Supplementary Figure S1).

These effects were likely mediated by IL6 binding to membrane-bound IL6 receptor (mbIL6R) in HL1 CMs, being observed in the absence of a soluble IL6 receptor (sIL6R). This is in line with similar previous reports in the same cell type [9], which was used to study the direct downregulation of connexin 40 and 43 induced by IL6 incubation.

**Figure 2:**
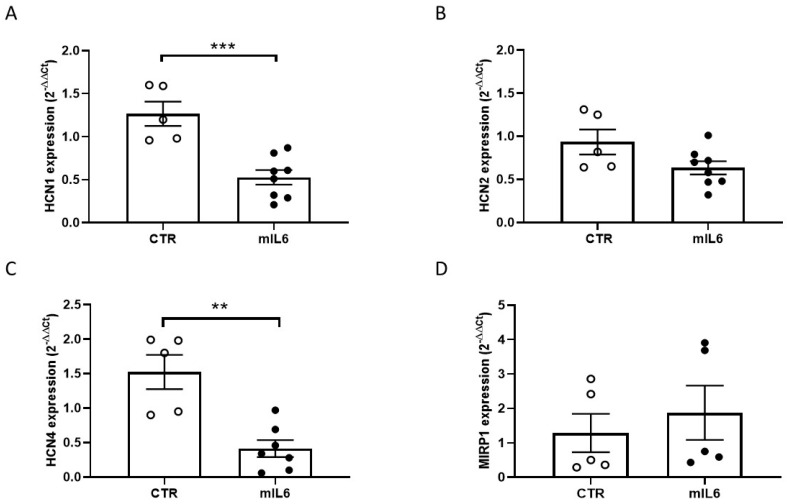
IL6 downregulates HCN channel transcripts in HL1 cells. HL1 cells were grown in normal medium with (mIL6) or without (CTR) mIL6 (50 ng/mL) for 24 h. Total RNA was isolated and qRT-PCR analysis was performed with specific primers targeted to HCN1 (**A**), 2 (**B**), 4 (**C**), MiRP1 (**D**), and GAPDH genes. GAPDH was used as an endogenous control. Data are expressed as mean ± standard error of 5–8 independent experiments. ** *p*  <  0.05, *** *p* < 0.01, *p* > 0.05 for HCN2/MiRP1 CTR vs. mIL6. Unpaired *t* test.

**Figure 3:**
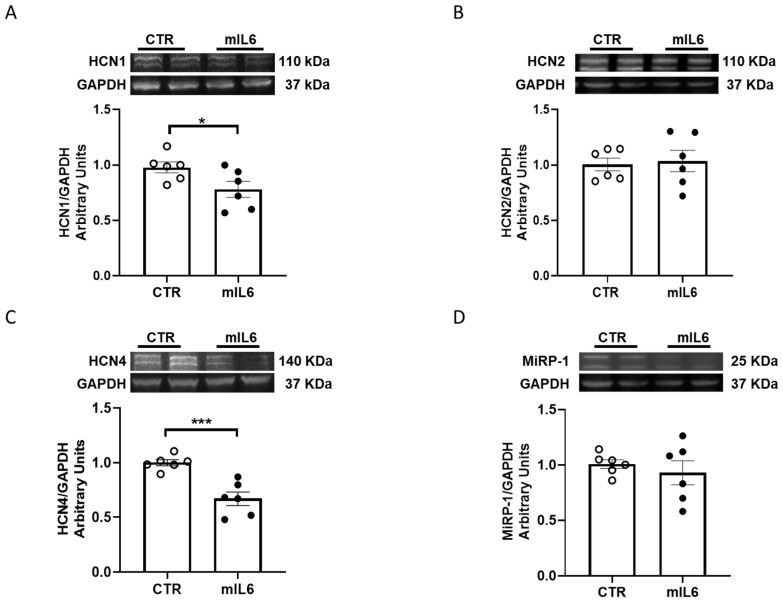
IL6 downregulates HCN channel proteins in HL1 cells. HL1 cells were grown in normal medium with (mIL6) or without (CTR) mIL6 (50 ng/mL) for 24 h. Proteins were analyzed by Western blot analysis. Panels show the representative immunoblots and the densitometric analysis of HCN1 (**A**), HCN2 (**B**), HCN4 (**C**), and MiRP1 (**D**) proteins. Data are expressed as mean ± standard error of 6 independent experiments. * *p*  <  0.05, *** *p*  <  0.001, *p* > 0.05 for HCN2/Mirp-1 CTR vs. mIL6. Unpaired *t* test.

Hence, in the same experimental conditions, we investigated the expression of HCN isoforms and the β subunit MIRP1 in HL1 CMs exposed to mIL6. Compared to untreated cells, a significant decrease in mRNA levels was observed for HCN1 and HCN4 isoforms, but not for HCN2 and MIRP1 (Figure 2). Despite the fact that large variability of HCN expression in HL1 CMs may have masked transcriptional effects due to the final tetrameric assembly of HCN channels, likely including different isoforms, the relevant message of our experiments is a general downregulation of HCN isoforms induced by IL6 exposure. Western blot assays confirmed that HCN1 and HCN4 protein levels were also significantly lowered in HL1 cells exposed to IL6 (Figure 3). 

### 2.2. Exposure to hIL6 Exerts Receptor-Dependent and Independent Effects on hiPSC-CMs

Due to the species-specific differences in mbIL6R expression and modulation in CMs, we aimed to verify the reproducibility of the IL6 signaling in human cells. Thus, we challenged CMs differentiated from human iPSC (hiPSC-CMs) with human IL6 (hIL6). Also in this case, a 30 min exposure to hIL6 triggered the pathway leading to STAT-3 phosphorylation, an effect completely abolished by pre-treatment with tocilizumab, the humanized mAb targeting the IL6 receptor (Supplementary Figure S2). Thus, in line with the expression of the mbIL6R by single cell analysis of human atrial CMs [12], the occurrence of the classical signaling associated with the mbIL6R seems to be similarly present in CMs from different mammalian species exposed to species-specific IL6. 

**Figure 4:**
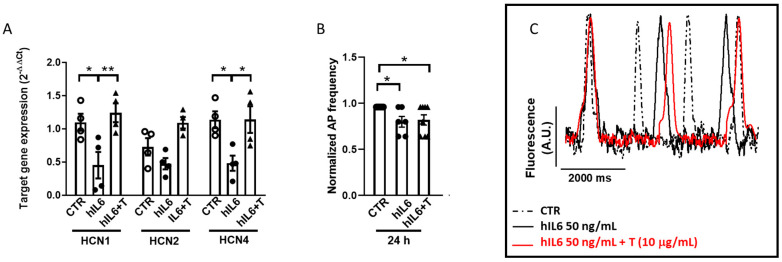
IL6 downregulates HCN channel expression and spontaneous action potential frequency in human iPSC-derived CMs. Total RNA was isolated from CMs exposed to hIL6 (50 ng/mL) with or without tocilizumab (T, 10 µg/mL) for 24 h (**A**); qRT-PCR analysis was performed with specific primers targeted to HCN1, 2, and 4; RLP19 was used as an endogenous control. Data are expressed as mean ± standard error of 4 independent experiments. * *p* < 0.05, ** *p* < 0.01, *p* > 0.05 for HCN2 CTR vs. hIL6, CTR vs. IL6 + T, hIL6 vs. IL6 + T. Ordinary two-way-ANOVA. Frequency of spontaneous APs recorded from CMs exposed to hIL6 (50 ng/mL) with or without tocilizumab (T, 10 µg/mL) for 24 h by a high-throughput system. Histogram (**B**) reports the normalized AP frequencies, expressed as mean ± standard error of 8 independent experiments, recorded at 24 h. Inset (**C**) shows representative recordings at 24 h. * *p* < 0.05. Ordinary one-way-ANOVA.

In the same experimental conditions, exposure to hIL6 for 24 h significantly decreased the mRNA levels of HCN1 and HCN4 but not of HCN2 isoforms (Figure 4A,B). Simultaneous incubation with tocilizumab was able to revert the effect of hIL6 at 24 h. Given the major role of the *f*-current in hiPSC-CMs automaticity [1,13], we searched for the functional consequences of hIL6 on HCN expression, e.g., the effect on cell beating rate. By employing a high-throughput optical mapping platform (MULTIPLE), we indeed found that the frequency of spontaneous action potentials (APs) in hiPSC-CMs exposed to hIL6 for 24 h was significantly reduced compared to CTR cells (Figure 4C). This effect was not prevented by simultaneous incubation of cells with 10 μg/mL tocilizumab; this raises the possibility that after a 24-h exposure to IL6, the time to recover the membrane expression of HCN (as well as other channels affected by IL6 and contributing to spontaneous beating rate) was not enough. No differences were observed in MiRP-1 expression in cells exposed to IL6 and IL6 + tocilizumab compared to control.

### 2.3. Acute Electrophysiological Effects of hIL6 in hiPSC-CMs

**Figure 5:**
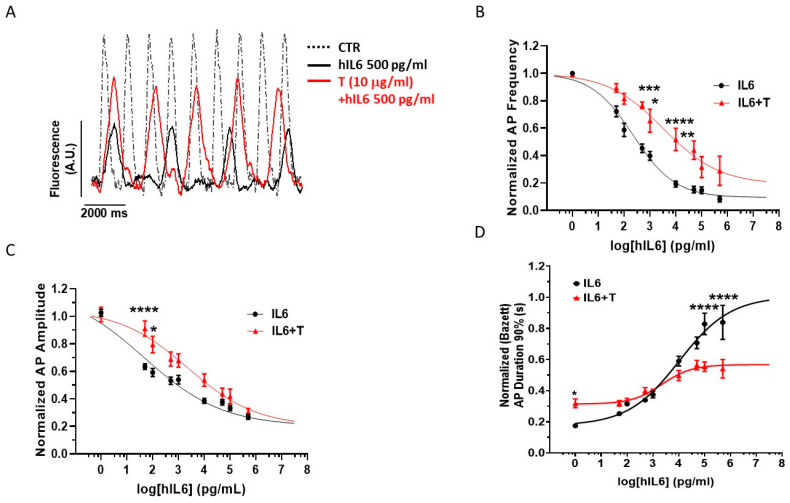
Acute hIL6 decreases spontaneous AP frequency and amplitude of human iPSC-derived CMs and increases its duration. CMs (differentiated for 39 days) were exposed to increasing hIL6 concentrations with or without tocilizumab (T, 10 µg/mL). Representative AP recordings are reported in (**A**). Normalized AP frequency ((**B**), *n* = 10–13), duration (90%, (**C**), *n* = 35–39), and amplitude ((**D**), *n* = 35–39) values, expressed as mean ± standard error of the mean, are plotted versus log of hIL6 concentration and fitted by Hill equation. AP durations at 90% of repolarization are normalized according to Bazett’s function. * *p* < 0.05, ** *p* < 0.01, *** *p* < 0.001, **** *p* < 0.0001 hIL6 vs. hIL6 + T. Two-way-ANOVA followed by Sidak’s multiple comparison test. A detailed statistical analysis of data in the graph is reported in Supplementary Table S1.

The observation made in Figure 4 prompted us to test the consequences of a rapid, acute exposure of *hiPSC-CMs* to hIL6; in fact, previous data obtained in in vivo and ex vivo animal cardiac models suggested that short-term exposure to IL6 exerts direct electrophysiological effects within a few minutes [14]. Indeed, in our experimental conditions, a rapid, dose-dependent electrophysiological response was observed in human iPSC-CMs challenged with hIL6 (Figure 5). AP frequency was reduced by hIL6 with an IC_50_ value of 0.22 ± 0.01 ng/mL (Figure 5B, black curve, *n* = 13), which was shifted leftward to 3.4 ± 0.04 ng/mL by simultaneous treatment with 10 μg/mL tocilizumab (Figure 5B, red curve, *n* = 10). Hill curve slope was −0.53 ± 0.055 and −0.40 ± 0.12 for hIL6 alone and hIL6 plus tocilizumab, respectively, suggesting a competitive behavior of the receptor antagonist. 

AP amplitude was reduced by hIL6 (Figure 5C, black curve) with an IC_50_ of 89.13 ± 1.20 pg/mL (*n* = 39) that was rightward shifted to 1412.54 ± 1.41 pg/mL (*n* = 35) by 10 μg/mL tocilizumab (Figure 5C, red curve). Hill curve slopes were −0.29 ± 0.018 and −0.33 ± 0.04 for hIL6 alone and hIL6 plus tocilizumab, respectively, still suggesting a competitive behavior. 

Corrected AP duration (measured at 90% of repolarization) was prolonged by hIL6 with an EC_50_ of 10.5 ± 2.34 ng/mL (Figure 5D, black curve, *n* = 39). Tocilizumab was also able to antagonize this effect, but a ≈50% drop in maximal inhibition and the increased Hill curve slope (IL6: 0.44 ± 0.11, IL6 + T: 0.75 ± 0.30) are indicative, in this case, of a predominantly noncompetitive mechanism.

### 2.4. hIL6 Directly Blunts HCN4-Mediated Current

**Figure 6:**
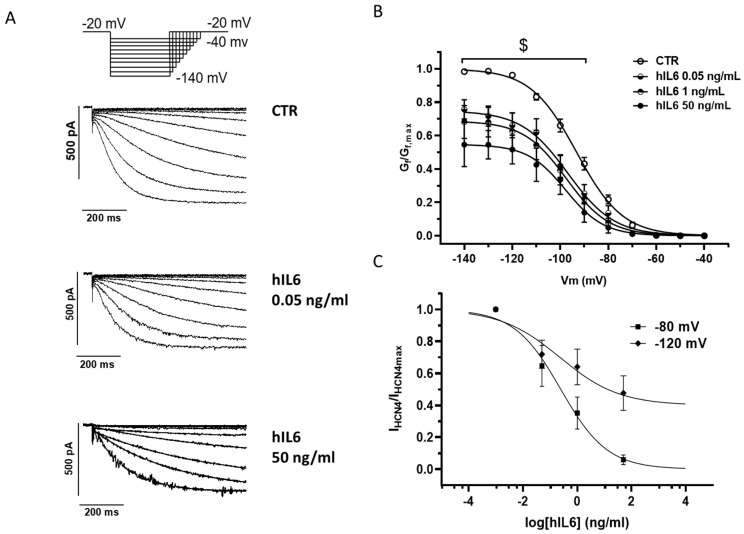
Acute hIL6 decreases HCN4-mediated current. Current was recorded in HEK cells stably expressing human HCN4 channels. (**A**) Voltage clamp protocol (**top panel**) and representative recordings (**bottom panels**) in control conditions (CTR) and after exposure to 0.05 and 50 ng/mL hIL6. (**B**) HCN4-current activation curves in control conditions (CTR) and after exposure to hIL6 (from 0.05 to 50 ng/mL). Values are expressed as mean of 4–7 independent experiment ± standard error of the mean. $ *p* < 0.05 CTR vs. hIL6 0.05 to 50 ng/mL. Two-way-ANOVA followed by Sidak’s multiple comparison test. A detailed statistical analysis of data in the graph is reported in Supplementary Table S2. (**C**) Concentration-current curve of normalized HCN4 current amplitude measured at −80 and −120 mV plotted versus log hIL6 concentration and fitted by Hill equation.

The acute decrease in spontaneous AP frequency by hIL6 led us to test whether hIL6 inhibits HCN4 current, as previously reported for hERG channels [15,16]. To this end, we carried out patch-clamp recordings in HEK cells stably expressing human HCN4 channel isoform: tracings (Figure 6A) and activation curves (Figure 6B) show that hIL6 blunts HCN4-mediated current in a concentration-dependent manner. The IC50 was 0.52 ± 0.23 and 0.61 ± 0.33 ng/mL (*n* = 5) at −80 and −120 mV, respectively (Figure 6C), with the maximum blockade by 50 ng/mL hIL6 being higher at −80 compared to −120 mV (Figure 6C). No relevant variations were observed for voltage dependence and kinetic properties of HCN4-mediated current (Supplementary Figure S3). Altogether, these results suggest that a direct and acute reduction in HCN-mediated currents may play a role in IL6-mediated reduction of AP spontaneous rate, and that the two mechanisms (the downregulation of HCN expression and the direct modulation of I_f_) may be additive or occur with a different time course, depending on the duration of the exposure. 

### 2.5. IL6 and HCN Expression in Human, Dilated Left Atrial Samples

A long-standing body of evidence exists of a correlation between inflammation and propensity to atrial myopathy [17], which may represent a risk for arrhythmogenesis in several conditions [18,19,20], including COVID-19 [21]. We reasoned that atrial dilation occurring in an overt pathological condition, such as mitral valve insufficiency, may represent a suitable clinical setting to test the hypothesis of a relationship between IL6 rise and HCN decrease [22]. Thus, we collected left atrial samples from 29 patients undergoing cardiac surgery for mitral valve (MV) replacement; atrial samples from non-diseased hearts explanted from donors served as control (age 46–50 years; male/female: 4/3). Supplementary Table S3 summarizes the MV patients’ characteristics and therapy. 

**Figure 7:**
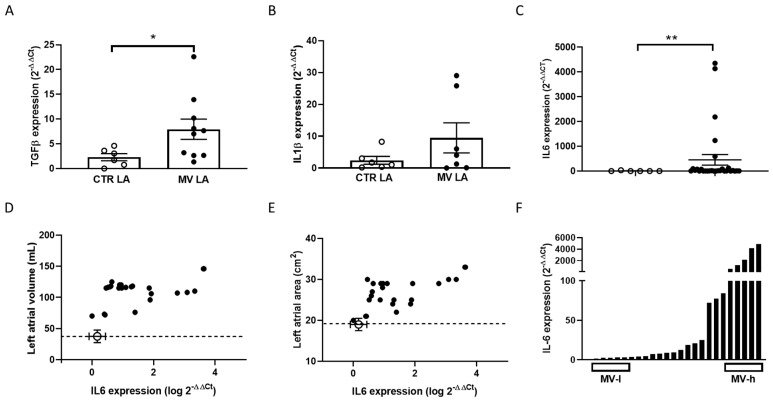
TGFβ, IL1β, and IL6 gene expression in human left atrial samples and correlations between echocardiographic left atrial volume or area and IL6 gene expression. TGFβ (**A**), IL1β (**B**), and IL6 (**C**) mRNA relative (GAPDH) amounts measured by RT-PCR in left atrial samples obtained from healthy donors (CTR LA, *n* = 7) and patients undergoing mitral valve replacement (MV LA, *n* = 10 for TGFβ, *n* = 7 for IL1β, and *n* = 29 for IL6) patients; * *p* < 0.05 unpaired *t* test, ** *p* < 0.01, non-parametric test followed by Mann–Whitney post-test. *p* > 0.05 for IL1β CTRLA vs. MVLA, unpaired *t* test. Left atrial maximal volume (**D**) or area (**E**) measured before surgery are plotted as a function of log IL6 expression detected in samples taken during cardiac surgery. Closed symbols represent values from MV patients, while open symbols refer to normal volume/area range values reported as reference in the literature [21] and expressed as a function of IL6 levels measured in our healthy donor atrial sample. Distribution of individual IL6 expression levels (**F**) measured in left atrial samples obtained from diseased patients undergoing cardiac surgery. Open bars indicate the samples with the lowest (MV-l, *n* = 5) and highest (MV-h, *n* = 5) IL6 expression, which were grouped in the following experiments.

We first assessed the expression of mRNA encoding for relevant cytokines TGFβ, IL1β, and IL6 by RT-PCR: both TGFβ and IL6 levels were significantly more elevated in samples obtained from patients undergoing MV replacement compared to control (CTR) samples (Figure 7A–C). Despite a large variability, log-transformed expression of IL6 levels displayed positive correlations when plotted against atrial maximal volume (Figure 7D, Pearson r = 0.049, *p* value= 0.0136) and area (Figure 7E, Pearson r = 0.066, *p* value= 0.0004). In addition, volume and area values were all above the normal ranges indicated by the open circle point and the dashed line. No correlation was found between IL6 level and age, sex, or other demographic parameters.

### 2.6. IL6 and HCN Expression in Left Atrial Samples

**Figure 8:**
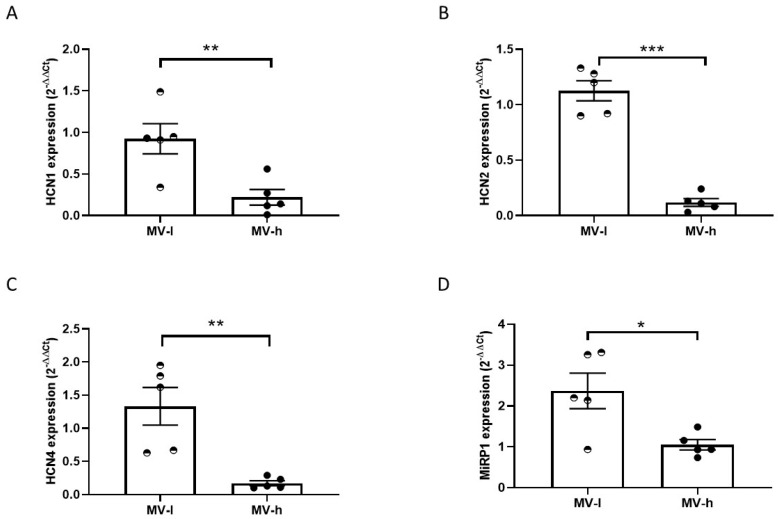
HCN 1, 2, 4, and MiRP1 gene expression in human left atrial samples with low and high IL6 levels. HCN1 (**A**), HCN2 (**B**), HCN4 (**C**), and MiRP1 (**D**) relative (RPL-19) mRNA amounts measured by RT-PCR in left atrial samples expressing low (MV-l, *n* = 5) or high (MV-h, *n* = 5) IL6 levels. * *p* < 0.05, ** *p* < 0.01, *** *p* < 0.001, unpaired *t* test.

In selected samples with the highest (MV-h) or with the lowest IL6 expression (MV-l) (Figure 7F), we assessed the expression of mRNA encoding for HCN channel subunits HCN1, HCN2, and HCN4 and the β-subunit MinK-related peptide 1 (MIRP1). The expression levels of HCN isoforms and MIRP1 were all significantly decreased in the MV-h with respect to MV-l samples (Figure 8A–D). 

## 3. Discussion

The hypothesis of the involvement of HCN channels in the electrophysiological abnormalities induced by cardiac cytokines stemmed from the evidence of the association between increased IL6 levels occurring in inflammatory and autoimmune diseases [14,23,24]. In particular, altered cardiac rhythm and conduction emerged in patients affected by COVID-19 acutely or subacutely [21], a condition where the so-called “cytokine storm” with high IL6 levels also plays a relevant role as a predictor of adverse outcome [21,25]. However, a role for IL6 in cardiac dysfunction, including propensity to arrhythmias, is not limited to systemic inflammatory diseases or viral infections.

Atrial inflammatory signals, including IL6 secreted by cardiac cells, promote structural and functional remodeling predisposing to arrhythmias [26]. Inflammation-induced atrial myopathy, often concomitant with metabolic diseases, likely precedes the occurrence of atrial fibrillation [17]. In keeping with this hypothesis, we observed that bioptic left atrial samples from patients with atrial dilation (and no fibrillation) undergoing MV replacement exhibit increased expression of cytokine levels. Moreover, mRNA levels of IL6 were positively related with left atrial area and diameter, a marker of atrial myopathy. We cannot infer that the tissue overexpression detected in our samples is exclusively of cardiomyocyte origin, although these cells represent the prevalent atrial tissue component, nor can we exclude that circulating IL6 may impact it as well. However, in our study, besides differences in atrial dilation, no major disparities were identified between MV-l and MV-h patients, since they are rather homogeneous for demography, cardiac pathology, co-morbidities, and medications (Supplementary Table S3). Given the low number of MV-l and MV-h patients in our study, larger studies are necessary to empower the statistical analysis and draw clear conclusions on the impact of atrial IL6 expression level on arrhythmic propension. 

Altered HCN channel function might contribute to the occurrence of brady- and tachy-arrhythmias reported in several conditions, including COVID-19-associated arrhythmogenic burden [27]. In the human heart, HCN4 lack of function is associated with sick sinus node syndrome, bradycardia, and atrio-ventricular block [28,29]. We observed that the highest IL6 expression in our human left atrial samples corresponds to the lowest expression of mRNA and protein levels of HCN isoforms. HCN isoforms and the *f*-current are constitutively present in atrial cardiomyocytes [1]; thus, atrial samples might be a suitable proxy of the consequences of IL6 sprouting to the nodes from the working surrounding tissue. Indeed, our recent unpublished observations detected IL6 overexpression in hiPSC-CMs and its release in the medium [30].

A mechanistic interpretation of our observations in human atrial samples comes from data obtained in murine atrial HL1 cells, where a reduction of HCN4 expression occurs after 24 h exposure to IL6. That this effect was mediated by the pathway downstream the membrane-bound IL6R receptor was suggested by the phosphorylation of STAT3 and translocation of NF-KB within 30 min in HL1. Likewise, the IL6 antagonist tocilizumab prevented hIL6-mediated phosphorylation of STAT3 in hiPSC-CMs. Tocilizumab also reverted the downregulation of HCN isoforms induced by 24 h exposure to IL6. Thus, the biological effect of IL6 observed in our experimental conditions appears to be achieved by the classical pathway, i.e., via the mbIL6R, which is present in human atrial CMs [12]. We cannot exclude that the trans-signaling pathway, involving the presence of a soluble IL6 receptor [24], which was not added in these experiments, may also play a role in vivo. Similarly, other pathways might contribute to the effect observed upon acute exposure to hIL6, i.e., the acute decline of spontaneous rate and I_f_ amplitude. This was not completely surprising: previous research has already reported the acute effect of IL6 on I_Kr_ in HEK293 and guinea pig cardiomyocytes [14,16]. The pathway downstream to IL6 stimulation interplays with other signals, such as CAMKII phosphorylation; pCAMKII, in turn, has been proven to phosphorylate several channels involved in cardiac electrogenesis [31]. This hypothesis deserves further investigation. 

Not surprisingly, due to the prominent effect of the *f*-current in CMs from pluripotent stem cells [1,32,33], downregulation of HCN mRNA after incubation with IL6 decreased the rate of iPSC-CMs spontaneous beating. Moreover, concentration-dependent (pg/mL to ng/mL) electrophysiological effects were also observed upon acute exposure to hIL6. The blunting effects on AP beating rate and amplitude were antagonized by tocilizumab in a competitive manner. These effects resemble those reported for *I*_Kr_ in HEK293 expressing hERG channels and in guinea pig cardiomyocytes [14,16].

At present, we can only infer the complex consequences of these effects on atrial (or nodal) properties; interestingly, a recent report showed that exposure of hiPSC-CMs to IL6 impairs intracellular calcium homeostasis [34]. These data not only support the direct stimulation mbIL-6R in human cardiomyocytes but also suggest that multiple mechanisms contribute to the altered electrophysiological properties of atrial cells. Further indirect evidence supporting a role for IL6-induced arrhythmias comes from a recent mouse model of atrial dilation, where blockade of IL6 trans-signaling attenuated the propensity to atrial fibrillation [20]. Notwithstanding the effect on atrial cells, where f-current plays a minor role (although constitutively present), more relevant consequences may occur in primary and subsidiary pacemakers. Cases of severe bradycardia were reported in COVID-19 patients [35], and cytokine storm in systemic inflammation may cause atrioventricular block [7] along with a general atrial electric remodeling [9]. Also, experimental inflammatory response was suggested to downregulate HCN4 and impair sinus node pacemaking in a mouse model of heart failure [11]. However, so far, no data are available on sinoatrial or atrioventricular cells concerning the effect of proinflammatory cytokines, and particularly IL6, a question that deserves further investigation.

## 4. Limitations and Conclusions

In our study, the combination of different cell types and models has obvious limitations and some strengths. Due to species-specific differences in the expression of HCN isoforms in different cell types, as well their changes depending on cardiac cell differentiation or disease [1], the quantitative effect of exposure to IL6 on HCN isoforms might differ, e.g., from murine to human cells. The different cell models allowed us to integrate different pieces of evidence that would have been difficult to be obtained in a single cell type or tissue. More important, in our view, is that the effect of IL6 on HCN isoforms, in particular HCN4 and HCN1, seems to be highly conserved from mouse to human cardiomyocytes.

In hiPSC-CMs, the reduction of AP frequency induced by 24 h exposure to hIL6 that is not prevented by tocilizumab is not obvious to explain. In fact, in Figure 4, we measured the transcript (mRNA level) of HCN channels and not the proteins due to the paucity of cells. Thus, it is possible that the time to recover the electrophysiological properties (i.e., either HCN or other channels contributing to spontaneous beating rate) was not enough. To try to get insight into this issue, we performed the experiments shown in Figure 5, where the acute effects of IL6 were tested. In this case, tocilizumab was able to revert, at least partially, the effect on AP frequency. We are aware that other channels, namely I_Kr_ and I_Ca_, are also affected by IL6 exposure and contribute to beating rate. Therefore, we cannot infer further the functional consequences of the downregulation of HCN and I_f_ decrease on the beating rate of cardiomyocytes. Such a demonstration was beyond the scope of the present work and is now investigated. However, we would like to strengthen the relevance of this finding: the HCN channel, which is indeed crucial for cardiac beating rate and conduction, as demonstrated by sound literature (reviewed by [1]), is affected by IL6.

In conclusion, our study identifies a direct link between IL6 levels and expression pattern of atrial HCN-channels, which suggests that targeting the inflammatory burden in the complex setting of atrial remodeling requires a deeper knowledge of the mechanisms, targets, and timing of specific anti-cytokine treatments.

## 5. Materials and Methods

### 5.1. HL1 Cell Culture and Viability Assay

HL1 cells (LOT: 2955183), obtained from Sigma-Aldrich (Schnelldorf, Germany), were plated on T25 flasks precoated overnight with 5 mg/mL fibronectin and 0.02% gelatin from bovine skin, as previously described [36,37,38]. Cells were maintained in Claycomb Medium supplemented with 10% fetal bovine serum 4 mL L-glutamine, 100 μM noradrenaline (norepinephrine) and 1% penicillin-streptomycin. The cultures were grown at 37 °C, 5% CO_2_, and 95% air at a relative humidity of approximately 95%. The medium was changed every 24 h. HL1 cells were passaged at a dilution 1:3 split ratio when they were 100% confluent (every 3–4 days). To do this, the cells were detached using a 5 min enzymatic dissociation with trypsin-EDTA. Digestion was stopped by adding medium and the sedimented cells were re-plated at a dilution of 1:3 to maintain cell culture or at 78,125 cells/cm^2^ density to perform experiments (passage 81–83). HL1 viability was determined by using a colorimetric assay based on 3-(4,5-dimethylthiazolyl-2)-2, 5-diphenyltetrazolium bromide (MTT). Briefly, HL1 cells were grown for 48 h in normal medium supplemented or not with mouse IL6 (50 ng/mL) and then were incubated with MTT labeling reagent (0.5 mg/mL in phosphate-buffered saline) at 37 °C for 3 h in the dark. After reagent removal, 100 µL 2-propanol was added to dissolve the formazan crystals included in the cells. Absorbance at 540 nm was read by a spectrophotometer. Each experimental sample was analyzed in triplicate. All reagents were purchased from Sigma-Aldrich (Schnelldorf, Germany).

### 5.2. hiPSC Culture and Cardiac Differentiation

Human-induced Pluripotent Stem Cells (hiPSCs, WTC11 line) were obtained from a healthy 30-33-year old male and were a gift of Dr. M.J. Pioner (University of Florence, Italy). They were maintained under feeder-free conditions in mTeSR Plus medium (Stem Cell Technologies, Vancouver, BC, Canada) on a Corning^®^ Matrigel matrix (Corning, NY, USA) and regularly passaged every 4–5 days. hiPSCs were differentiated onto a Matrigel matrix by a monolayer-directed differentiation protocol using the cardiac PSC Cardiomyocyte Differentiation Kit (Life Technologies, Thermo Fisher scientific, Carlsbad, CA, USA) following the manufacturer’s instructions, as previously described [38,39,40,41]. Briefly, hiPSC colonies with 70–80% confluency were chemically dissociated using 1× TrypLE (Life Technologies, Thermo Fisher scientific, Carlsbad, CA, USA) suspended into mTeSR Plus with 5 μM ROCK inhibitor (Y27632, Stem Cell Technologies, Vancouver, BC, Canada) and seeded as single cells onto Matrigel-coated wells of a 24-wells plate at a cell density of 80,000 cells/well. At 70% confluency (2–3 days), the cardiac induction was started by changing the culture medium to Cardiomyocyte Differentiation Medium A (day 0). After two days, Medium A was replaced with Medium B and on the following 2 days with Medium C for final CM differentiation. hiPSC-CMs were fed every other day with Medium C until spontaneously beating monolayers appeared (day 8–10). At day 12, Medium C was replaced with RPMI plus B27 supplement (Life Technologies, Thermo Fisher scientific, Carlsbad, CA, USA), which was refreshed every 2 days, for further hiPSC-CMs maturation. For experiments, hiPSC-CMs (passage 64–73) were used on day 30–39 of maturation. 

### 5.3. Culture of HEK293 Expressing HCN4 Channel

Human embryonic kidney cells (HEK293 cells DSMZ, Braunschweig, Germany), transfected with human HCN4 (hHCN4) cDNA (provided by Prof. M. Biel, University of München, Germany), were cultured in T25 flasks and incubated at 37 °C with 5% CO_2_, as previously described [42]. Cells were maintained in Dulbecco’s modified Eagle’s medium, High Glucose (DMEM + GlutaMax-Ix1), supplemented with 10% fetal bovine serum, 100 units/mL penicillin, 100 µg/mL streptomycin, 200 µg/mL geneticin (G418). At confluence, cells were detached by enzymatic dissociation with trypsin-EDTA; digestion was stopped by adding complete medium. After centrifugation at 1200 rpm for 5 min, the sedimented cells were either replated or used for electrophysiological measurements. All reagents were purchased from Thermo Fisher Scientific, Carlsbad, CA, USA. For HCN4-current (I_HCN4_) recordings, HEK293 cells (passage 25–28) were dissociated with diluted trypsin in PBS solution (1:10) and then were incubated for 2–3 h at room temperature in Tyrode’s solution (see “Section 5.10”), which was supplemented with CaCl_2_ (300 μM, Sigma-Aldrich, Schnelldorf, Germany) and Bovine Serum (10 mg/mL; Sigma-Aldrich, Schnelldorf, Germany). 

### 5.4. Human Atrial Samples

The research design complied with the Declaration of Helsinki, and the study was approved by the local ethics committee (#STAF15, Comitato Etico Regione Toscana—Area Vasta Sud Est). All subjects (>18 years) from Le Scotte University Hospital (Siena, Italy) gave a written informed consent for participation. Left atrial samples were obtained during cardiac valve surgery. Control left atrial samples were obtained from undiseased organ donors whose hearts were explanted to obtain pulmonary and aortic valves eligible for transplant (University of Szeged). Before explantation, these organ donor patients did not receive medication except dobutamine, furosemide, and plasma expanders. All experimental protocols were approved by the Albert Szent-Gyorgyi Medical University Ethical review Board (No. 51-57/1997 OEj). Samples were quickly frozen in liquid nitrogen and stored at −80 °C until analysis. Characteristics of patients are reported in Table S3. 

### 5.5. Reverse Transcription-Quantitative Polymerase Chain Reaction

Total mRNA was isolated from frozen human atrial samples, HL1 cells, and hiPSC-CM cultures using the QIAzol Lysis Reagent (QIAGEN S.r.l., Milano, Italy). Subsequently, complementary DNA (cDNA) was synthesized from 1 μg of total RNA using iScript™ cDNA Synthesis kit (Bio-Rad Laboratories S.r.l., Milan, Italy). All these steps were performed according to the manufacturer’s instructions. Real-time quantitative PCR (Q-PCR) was performed using iTaq™ SYBR^®^ Green Universal Supermix (Bio-Rad Laboratories S.r.l., Milan, Italy) and predesigned primers assays for the following genes: Transforming Growth Factor β (TGFβ), Interleukin 1β (IL1β), Interleukin 6 (IL6), hyperpolarization-activated cyclic nucleotide-gated channel 1, 2, 4 (HCN1, HCN2, HCN4), and MinK-related peptide 1 (MiRP1 or KCNE2) (Bio-Rad Laboratories S.r.l., Milan, Italy). Relative quantification of the mRNA level for the different genes was determined with the Bio-Rad CFX Maestro 1.1 software (4.1.2433.1219, Bio-Rad Laboratories S.r.l., Milan, Italy) using the comparative method (∆∆Ct).

### 5.6. Western Blot Analysis

Protein lysates were recovered from plated HL1 cells or hiPSC-derived CMs using a lysis buffer containing 50mM Tris HCl (pH 8), 150 mM NaCl, 1mM EDTA, 0.1% *w*/*v* sodium dodecyl sulfate, and a protease and phosphatase inhibitor cocktail (Thermo Fisher Scientific, Carlsbad, CA, USA). Total protein levels were quantified using the Pierce Protein Assay/bicinchoninic acid (Thermo Fisher Scientific, Carlsbad, CA, USA). Subsequently, 20 µg of proteins were separated on 4–20% SDS-PAGE (Thermo Fisher Scientific, Carlsbad, CA, USA) and transferred into PVDF membranes (60 min at 398 mA) using standard procedures. Blots were incubated overnight at 4 °C with a specific antibody against HCN1 (rabbit, #APC-056, Lot APC056AG0740, Alomone Labs, Jerusalem, Israel), HCN2 (rabbit, #APC-030, Lot APC030AG0940, Alomone Labs, Jerusalem, Israel), HCN4 (rabbit, #APC-052, Lot APC052AG11120, Alomone Labs, Jerusalem, Israel), MiRP1 (rabbit, #APC-054, Lot AN-03, Alomone Labs, Jerusalem, Israel), *p*-STAT3Tyr705 (mouse, #4113S, Lot 6, Cell Signaling Technology, Leiden, The Netherland), STAT3 (rabbit, #4904S, Lot 7, Cell Signaling Technology, Leiden, The Netherland), and GAPDH (rabbit, #5174S, Lot 8, Cell Signaling Technology, Leiden, The Netherland). Primary antibodies were diluted in PBS containing 5% bovine serum albumin or 5% non-fat dry milk and 0.05% Tween. The antigen–antibody complexes were visualized using appropriate secondary antibodies (Goat anti-rabbit #7074S, Lot 31, Cell Signaling Technology, Leiden, The Netherland; Horse anti-mouse #7076S, Lot 38, Cell Signaling Technology, Leiden, The Netherland; diluted 1:3000 in PBS containing 5% bovine serum albumin or 5% non-fat dry milk and 0.05% Tween) and left for 1 h at room temperature. Blots were then extensively washed with PBS containing 0.1% Tween and developed using an enhanced chemiluminescence detection system (Thermo Fisher Scientific, Carlsbad, CA, USA). Exposure and developing time were standardized for all blots. Densitometric analysis of acquired images was performed using the public domain ImageJ software (version 1.53n, NIH, Bethesda, MA, USA). When multiple bands were observed, the band corresponding approximately to the molecular weight reported in the figures was analyzed. Results represent the mean ± standard error of the mean of different gels and are expressed as arbitrary units (AUs), consisting of the ratio between the levels of target protein and GAPDH. All reagents, unless otherwise specified, were purchased from Merck Life Science S.r.l. (Milano, Italy).

### 5.7. Immunofluorescence

Hl1 cells were seeded on coverslips, treated, and then fixed in 4% paraformaldehyde for 10 min. Cells were then permeabilized with PBS containing 0.1% Tryton X-100 and incubated in blocking solution (0.1% Tryton X-100 and Immobilon Block—FL, Merk Millipore, Milan, Italy) for 1 h at room temperature. Then, cells were incubated with the primary antibody (NF-kB, rabbit, #8242S, Lot 9, 1:200, Cell signaling Technology, Danvers, MA, USA) for 1h at room temperature, washed in PBS, and incubated with the secondary antibody (Anti-Rabbit IgG (whole molecule), F(ab′)2 fragment–Cy3 antibody produced in sheep, #C2306, Lot SLBK3506V, Sigma-Aldrich, Schnelldorf, Germany) for 1 h at room temperature. Cell nuclei were labeled with DAPI 1 µg/mL (#D9542, Source 13190309, Batch 0000116964, Sigma-Aldrich, Schnelldorf, Germany) for 20 min at room temperature. After washes in PBS, the coverslips were put on cover slides to be observed with an inverted confocal microscope (Leica SP8 Confocal Microscope, Leica Microsystems, Wetzlar, Germany). To assess NF-κB/DAPI colocalization, Mander’s coefficient (M1) was determined through ImageJ software (version 1.53n, NIH, Bethesda, MA, USA). Data are expressed as means ± standard errors of the mean. Statistical significance was set at a value of *p* < 0.05 and was determined by *t*-test. 

### 5.8. Action Potential Recordings from hiPSC-CMs

The spontaneous electrical activity of hiPSC-CMs was recorded using the High-Throughput MULTIPLE system, as previous described [38,41]. Monolayers of hiPSC-CMs were incubated with a near-infrared voltage-sensitive dye (VSD) di-4-ANBDQPQ [43] for 9 min and then washed with dye-free Tyrode’s solution (see “Section 5.10”) containing 1.8 mM CaCl_2_ at 37 °C. They were placed on the High-Throughput MULTIPLE microscope stage and maintained at a constant temperature of 37 °C during recordings. Cell monolayers were illuminated with red LED (SOLIS-623C, Thorlabs, Newton, NJ, USA) followed by a band-pass filter (625PB50, Omega optical, Brattleboro, VT, USA). The fluorescence signal was collected in the forward direction using a camera lens (MVL12M43, Thorlabs, Newton, NJ, USA) placed in front of a sCMOS camera (ORCA-Flash 4.0 V3, Hamamatsu Photonics, Hamamatsu City, Japan) operating at a frame rate of 100 Hz. A long-pass filter (LP700; Omega optical, Brattleboro, VT, USA) was placed in front of the camera lens [38]. 

### 5.9. Patch Clamp Recordings

Patch-clamp recordings were performed in the whole-cell configuration [44] using an inverted microscope (Zeiss Axiovert 135, Malente, Germany, Nikon Diaphot TMD, Tokyo, Japan). The experiments were performed with a patch amplifier (Axopatch-200B, Axon Instruments, Union, CA, USA) and signals were digitalized with Digidata 1440 A (Axon Instruments, Union, CA, USA) and viewed online on the computer screen. Experimental control, data acquisition, and preliminary analysis were performed by means of the integrated software package pClamp (Axon Instruments, Union, CA, USA). Patch-clamp pipettes were prepared from glass capillary tubes (Harvard Apparatus Ltd., Kent, UK) by means of a two-stage horizontal puller (model *p*-87; Sutter Instrument, Novato, CA, USA). Pipette resistance was 4–6 MΩ when filled with the internal solution (see “Section 5.10”). HEK293 cells were continuously perfused with Tyrode’s solution supplemented with 1.8 mM CaCl_2_ or modified Tyrode’s solution (see “Section 5.10”) using a gravity-fed perfusion system. The temperature was maintained in the range of 37 ± 1 °C. I_HCN4_ was evoked from a holding potential of −20 mV to more negative voltage steps of −40 to −140 in 10 mV increments [42].

### 5.10. Solutions

Tyrode’s solution (mM): D-(+)-glucose 10, NaCl 140, KCl 5.4, MgCl_2_ 1.2, Hepes 5.0, adjusted to pH 7.3with NaOH. Modified Tyrode’s solution for I_HCN4_ recording: Tyrode’s solution supplemented with KCl 19.6 mM. Intracellular solution (mM): K-aspartate 130, Na_2_-ATP 5, MgCl_2_ 2, EGTA 11, CaCl_2_ 5, Hepes 10, adjusted to pH 7.2 with KOH. Drug solutions—murine and human IL6 (Sigma-Aldrich, Schnelldorf, Germany; 100 μg/mL) and tocilizumab (MedChemExpress, Monmouth Junction, NJ, USA; 10 mg/mL)were diluted in the different experimental solutions to reach the desired final concentration (IL6: 50 ng/mL, as described in [31], tocilizumab 10 μg/mL).

### 5.11. Data and Statistical Analyses

Relative quantification of the mRNA level for the different genes was determined with the Bio-Rad CFX Maestro software 1.1 (version 4.1.2433.1219, Bio-Rad Laboratories S.r.l., Milan, Italy), using the comparative method (∆∆Ct).

Fluorescent signals obtained using the High-Throughput system were associated with regions of interest (ROI) and were photo-bleached, corrected, normalized, and temporally filtered using LabVIEW 2021_21.5(National Instruments, Austin, TX, USA), Fiji-ImageJ 1.53n (NIH, Bethesda, MA, USA), nd OriginLab 2023 (Northampton, MA, USA) software [38]. OriginLab 2023 software was used to analyse action potential amplitude (APA) and duration at 90% of the repolarization phase (APD_90_). To evaluate the influence of rate variability on APD_90_, we used correction algorithms [45], as previously performed for AP measurements in human pluripotent-derived CMs [46,47]. Amongst the different algorithms, we used Bazett’s Formula (1):

(1) $${APD}_{90c}=\frac{{APD}_{90}}{\sqrt{F}}$$

where APD90c (s) is the corrected APD_90_ and F (s) is the frequency of spontaneous AP.

I_HCN4_ amplitudeobtained by patch-clamp recording was measured by fitting with the Clampfit program (pClamp 10.7, Axon Instruments, Union, CA, USA), the time-dependent component of current with a mono-exponential function. The specific conductance of each cell was determined according to Equation (2):

(2) $$G_{HCN}=\frac{I}{\left(Vm-Vrev\right)}$$

where G_HCN_ is the conductance (pS/pF) calculated at membrane potential V_m_, I is the current density (pA/pF), and V_rev_ is the current reversal potential calculated from the analysis of tail currents [44]. 

Activation curves of I_HCN4_ were fitted to Boltzmann’s Equation (3):

(3) $$G_{HCN}=\frac{Gmax}{\left(1+e^{\frac{V0.5-Vm}{k}}\right)}$$

where V_m_ (mV) is the test membrane potential, V_0.5_ (mV) is the fitted potential for half-maximal activation, and k (mV) is the slope factor of the activation curve. 

Concentration–effect curves obtained with different human IL6 concentrations (0.05–50 ng/mL) were fitted to a Hill distribution: (Equation (4)):

(4) $$E=E_{max}\frac{x^{n}}{k^{n}+x^{n}}$$

where E_max_ is the maximum effect, k is the concentration for half-maximal inhibition (IC_50_), x is the drug concentration, and *n* is the Hill coefficient. 

Statistical analysis of data and curve fitting were performed by using GRAPHPAD PRISM (version 8.0; GRAPHPAD Software, San Diego, CA, USA). Statistical significance was set at a value of *p* < 0.05 and was determined by t-test or one or two-way ANOVA as appropriate. All data are reported as mean ± standard error of the mean.

## Data Availability

Data are contained within the article and Supplementary Materials.

## Supplementary Materials

The following supporting information can be downloaded at: https://www.mdpi.com/article/10.3390/ijms252212212/s1.

## References

[1] Sartiani L., Mannaioni G., Masi A., Novella Romanelli M., Cerbai E. (2017). The Hyperpolarization-Activated Cyclic Nucleotide-Gated Channels: From Biophysics to Pharmacology of a Unique Family of Ion Channels. Pharmacol. Rev..

[2] Oniani T., Vinnenberg L., Chaudhary R., Schreiber J.A., Riske K., Williams B., Pape H.C., White J.A., Junker A., Seebohm G. (2022). Effects of Axonal Demyelination, Inflammatory Cytokines and Divalent Cation Chelators on Thalamic HCN Channels and Oscillatory Bursting. Int. J. Mol. Sci..

[3] Rivolta I., Binda A., Masi A., DiFrancesco J.C. (2020). Cardiac and neuronal HCN channelopathies. Pflug. Arch..

[4] Resta F., Micheli L., Laurino A., Spinelli V., Mello T., Sartiani L., Di Cesare Mannelli L., Cerbai E., Ghelardini C., Romanelli M.N. (2018). Selective HCN1 block as a strategy to control oxaliplatin-induced neuropathy. Neuropharmacology.

[5] Ridker P.M., Libby P., MacFadyen J.G., Thuren T., Ballantyne C., Fonseca F., Koenig W., Shimokawa H., Everett B.M., Glynn R.J. (2018). Modulation of the interleukin-6 signalling pathway and incidence rates of atherosclerotic events and all-cause mortality: Analyses from the Canakinumab Anti-Inflammatory Thrombosis Outcomes Study (CANTOS). Eur. Heart J..

[6] Ridker P.M. (2019). Anticytokine Agents: Targeting Interleukin Signaling Pathways for the Treatment of Atherothrombosis. Circ. Res..

[7] Lazzerini P.E., Acampa M., Cupelli M., Gamberucci A., Srivastava U., Nanni C., Bertolozzi I., Vanni F., Frosali A., Cantore A. (2021). Unravelling Atrioventricular Block Risk in Inflammatory Diseases: Systemic Inflammation Acutely Delays Atrioventricular Conduction via a Cytokine-Mediated Inhibition of Connexin43 Expression. J. Am. Heart Assoc..

[8] Lazzerini P.E., Accioli R., Acampa M., Zhang W.H., Verrengia D., Cartocci A., Bacarelli M.R., Xin X., Salvini V., Chen K.S. (2022). Interleukin-6 Elevation Is a Key Pathogenic Factor Underlying COVID-19-Associated Heart Rate-Corrected QT Interval Prolongation. Front. Cardiovasc. Med..

[9] Lazzerini P.E., Laghi-Pasini F., Acampa M., Srivastava U., Bertolozzi I., Giabbani B., Finizola F., Vanni F., Dokollari A., Natale M. (2019). Systemic Inflammation Rapidly Induces Reversible Atrial Electrical Remodeling: The Role of Interleukin-6-Mediated Changes in Connexin Expression. J. Am. Heart Assoc..

[10] Douedi S., Mararenko A., Alshami A., Al-Azzawi M., Ajam F., Patel S., Douedi H., Calderon D. (2021). COVID-19 induced bradyarrhythmia and relative bradycardia: An overview. J. Arrhythm..

[11] Kahnert K., Soattin L., Mills R.W., Wilson C., Maurya S., Sorrentino A., Al-Othman S., Tikhomirov R., van de Vegte Y.J., Hansen F.B. (2024). Proteomics couples electrical remodelling to inflammation in a murine model of heart failure with sinus node dysfunction. Cardiovasc. Res..

[12] Karlsson M., Zhang C., Méar L., Zhong W., Digre A., Katona B., Sjöstedt E., Butler L., Odeberg J., Dusart P. (2021). A single-cell type transcriptomics map of human tissues. Sci. Adv..

[13] Giannetti F., Benzoni P., Campostrini G., Milanesi R., Bucchi A., Baruscotti M., Dell’Era P., Rossini A., Barbuti A. (2021). A detailed characterization of the hyperpolarization-activated “funny” current (*I*_f_) in human-induced pluripotent stem cell (iPSC)–derived cardiomyocytes with pacemaker activity. Pflug. Arch..

[14] Cupelli M., Ginjupalli V.K.M., Chen L., Capecchi P.L., Lazzerini P.E., Boutjdir M., El-Sherif N. (2023). Contribution of cytokine-mediated prolongation of QTc interval to the multi-hit theory of Torsade de Pointes. Biochem. Biophys. Res. Commun..

[15] Lazzerini P.E., Laghi-Pasini F., Bertolozzi I., Morozzi G., Lorenzini S., Simpatico A., Selvi E., Bacarelli M.R., Finizola F., Vanni F. (2017). Systemic inflammation as a novel QT-prolonging risk factor in patients with torsades de pointes. Heart.

[16] Aromolaran A.S., Srivastava U., Alí A., Chahine M., Lazaro D., El-Sherif N., Capecchi P.L., Laghi-Pasini F., Lazzerini P.E., Boutjdir M. (2018). Interleukin-6 inhibition of hERG underlies risk for acquired long QT in cardiac and systemic inflammation. PLoS ONE.

[17] Packer M. (2020). Characterization, Pathogenesis, and Clinical Implications of Inflammation-Related Atrial Myopathy as an Important Cause of Atrial Fibrillation. J. Am. Heart Assoc..

[18] Aviles R.J., Martin D.O., Apperson-Hansen C., Houghtaling P.L., Rautaharju P., Kronmal R.A., Tracy R.P., Van Wagoner D.R., Psaty B.M., Lauer M.S. (2003). Inflammation as a risk factor for atrial fibrillation. Circulation.

[19] Chen Y.C., Voskoboinik A., Gerche A., Marwick T.H., McMullen J.R. (2021). Prevention of Pathological Atrial Remodeling and Atrial Fibrillation: JACC State-of-the-Art Review. J. Am. Coll. Cardiol..

[20] Li X., Wu X., Chen X., Peng S., Chen S., Zhou G., Wei Y., Lu X., Zhou C., Ye Y. (2023). Selective blockade of interleukin 6 trans-signaling depresses atrial fibrillation. Heart Rhythm.

[21] Guzik T.J., Mohiddin S.A., Dimarco A., Patel V., Savvatis K., Marelli-Berg F.M., Madhur M.S., Tomaszewski M., Maffia P., D’Acquisto F. (2020). COVID-19 and the cardiovascular system: Implications for risk assessment, diagnosis, and treatment options. Cardiovasc. Res..

[22] Meyer T.E., Chen K., Parker M.W., Shih J., Rahban Y. (2023). Perspectives on Secondary Mitral Regurgitation in Heart Failure. Curr. Heart Fail. Rep..

[23] Lazzerini P.E., Capecchi P.L., Laghi-Pasini F. (2017). Systemic inflammation and arrhythmic risk: Lessons from rheumatoid arthritis. Eur. Heart J..

[24] Hunter C.A., Jones S.A. (2015). IL-6 as a keystone cytokine in health and disease. Nat. Immunol..

[25] Ruan Q., Yang K., Wang W., Jiang L., Song J. (2020). Clinical predictors of mortality due to COVID-19 based on an analysis of data of 150 patients from Wuhan, China. Intensive Care Med..

[26] Ihara K., Sasano T. (2022). Role of Inflammation in the Pathogenesis of Atrial Fibrillation. Front. Physiol..

[27] Coromilas E.J., Kochav S., Goldenthal I., Biviano A., Garan H., Goldbarg S., Kim J.H., Yeo I., Tracy C., Ayanian S. (2021). Worldwide Survey of COVID-19-Associated Arrhythmias. Circ. Arrhythm. Electrophysiol..

[28] Boyett M.R., Yanni J., Tellez J., Bucchi A., Mesirca P., Cai X., Logantha S.J.R.J., Wilson C., Anderson C., Ariyaratnam J. (2021). Regulation of sinus node pacemaking and atrioventricular node conduction by HCN channels in health and disease. Prog. Biophys. Mol. Biol..

[29] Baruscotti M., Bucchi A., Viscomi C., Mandelli G., Consalez G., Gnecchi-Rusconi T., Montano N., Casali K.R., Micheloni S., Barbuti A. (2011). Deep bradycardia and heart block caused by inducible cardiac-specific knockout of the pacemaker channel gene Hcn4. Proc. Natl. Acad. Sci. USA.

[30] Guzzolino E., Balducci V., Allegro G., Spinelli V., Ninu A., Lo Presti F., Sacconi L., Cameli M., Stefano P.L., Sartiani L. Uncovering a novel regulatory circuit among mir-182, interleukin-6 and HCN4: New perspectives in cardiac congenital arrhythmias and human atrial Fibrillation. Proceedings of the 40 EWGCCE.

[31] Zhao L., Cheng G., Jin R., Afzal M.R., Samanta A., Xuan Y.T., Girgis M., Elias H.K., Zhu Y., Davani A. (2016). Deletion of Interleukin-6 Attenuates Pressure Overload-Induced Left Ventricular Hypertrophy and Dysfunction. Circ. Res..

[32] Sartiani L., Bettiol E., Stillitano F., Mugelli A., Cerbai E., Jaconi M.E. (2007). Developmental changes in cardiomyocytes differentiated from human embryonic stem cells: A molecular and electrophysiological approach. Stem Cells.

[33] Bosman A., Sartiani L., Spinelli V., Del Lungo M., Stillitano F., Nosi D., Mugelli A., Cerbai E., Jaconi M. (2013). Molecular and functional evidence of HCN4 and caveolin-3 interaction during cardiomyocyte differentiation from human embryonic stem cells. Stem Cells Dev..

[34] Lin A.E., Bapat A.C., Xiao L., Niroula A., Ye J., Wong W.J., Agrawal M., Farady C.J., Boettcher A., Hergott C.B. (2024). Clonal Hematopoiesis of Indeterminate Potential With Loss of Tet2 Enhances Risk for Atrial Fibrillation Through Nlrp3 Inflammasome Activation. Circulation.

[35] Amaratunga E.A., Corwin D.S., Moran L., Snyder R. (2020). Bradycardia in Patients With COVID-19: A Calm Before the Storm?. Cureus.

[36] Claycomb W.C., Lanson N.A., Stallworth B.S., Egeland D.B., Delcarpio J.B., Bahinski A., Izzo N.J. (1998). HL-1 cells: A cardiac muscle cell line that contracts and retains phenotypic characteristics of the adult cardiomyocyte. Proc. Natl. Acad. Sci. USA.

[37] Sartiani L., Bochet P., Cerbai E., Mugelli A., Fischmeister R. (2002). Functional expression of the hyperpolarization-activated, non-selective cation current I(f) in immortalized HL-1 cardiomyocytes. J. Physiol..

[38] Credi C., Balducci V., Munagala U., Cianca C., Bigiarini S., de Vries A.A.F., Loew L.M., Pavone F.S., Cerbai E., Sartiani L. (2021). Fast Optical Investigation of Cardiac Electrophysiology by Parallel Detection in Multiwell Plates. Front. Physiol..

[39] Dell’Era P., Benzoni P., Crescini E., Valle M., Xia E., Consiglio A., Memo M. (2015). Cardiac disease modeling using induced pluripotent stem cell-derived human cardiomyocytes. World J. Stem Cells.

[40] Pioner J.M., Santini L., Palandri C., Martella D., Lupi F., Langione M., Querceto S., Grandinetti B., Balducci V., Benzoni P. (2019). Optical Investigation of Action Potential and Calcium Handling Maturation of hiPSC-Cardiomyocytes on Biomimetic Substrates. Int. J. Mol. Sci..

[41] Balducci V., Credi C., Sacconi L., Romanelli M.N., Sartiani L., Cerbai E. (2021). The HCN channel as a pharmacological target: Why, where, and how to block it. Prog. Biophys. Mol. Biol..

[42] Del Lungo M., Melchiorre M., Guandalini L., Sartiani L., Mugelli A., Koncz I., Szel T., Varro A., Romanelli M.N., Cerbai E. (2012). Novel blockers of hyperpolarization-activated current with isoform selectivity in recombinant cells and native tissue. Br. J. Pharmacol..

[43] Matiukas A., Mitrea B.G., Qin M., Pertsov A.M., Shvedko A.G., Warren M.D., Zaitsev A.V., Wuskell J.P., Wei M.D., Watras J. (2007). Near-infrared voltage-sensitive fluorescent dyes optimized for optical mapping in blood-perfused myocardium. Heart Rhythm.

[44] Cerbai E., Barbieri M., Mugelli A. (1994). Characterization of the hyperpolarization-activated current, I(f), in ventricular myocytes isolated from hypertensive rats. J. Physiol..

[45] Luo S., Michler K., Johnston P., Macfarlane P.W. (2004). A comparison of commonly used QT correction formulae: The effect of heart rate on the QTc of normal ECGs. J. Electrocardiol..

[46] Doss M.X., Di Diego J.M., Goodrow R.J., Wu Y., Cordeiro J.M., Nesterenko V.V., Barajas-Martínez H., Hu D., Urrutia J., Desai M. (2012). Maximum diastolic potential of human induced pluripotent stem cell-derived cardiomyocytes depends critically on I(Kr). PLoS ONE.

[47] Bosman A., Letourneau A., Sartiani L., Del Lungo M., Ronzoni F., Kuziakiv R., Tohonen V., Zucchelli M., Santoni F., Guipponi M. (2015). Perturbations of heart development and function in cardiomyocytes from human embryonic stem cells with trisomy 21. Stem Cells.

