# Supplementary material for: Interleukin-6 Modulates the Expression and Function of HCN Channels: A Link Between Inflammation and Atrial Electrogenesis
Source: Int J Mol Sci. 2024 Nov 14;25(22):12212. doi: 10.3390/ijms252212212 (PMC11594737; doi:10.3390/ijms252212212)
Supplement: Supplementary file 1 [file ijms-25-12212-s001.zip › ijms-3287271-supplementary.pdf]

Supplementary materials and methods

Figure S1: IL6 does not alter HL1 cell viability. HL1 cells were grown for 48 h in normal medium supplemented or not with mouse IL6 (50 ng/mL). Cell viability was measured by MTT assay.

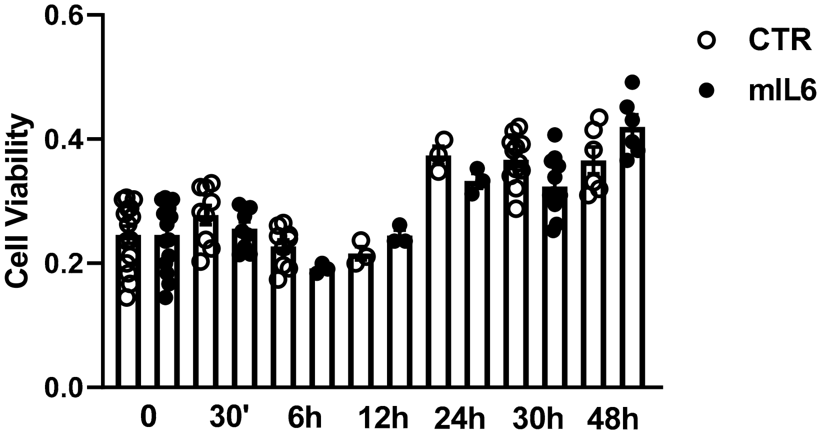

Figure S2: hIL6 induces STAT-3 phosphorylation in human iPSC-derived CMs. CMs (differentiated for 30 days) were exposed to hIL6 (50ng/mL) with or without Tocilizumab (T, 10 ug/mL) for 30 minutes. Western blot analysis was performed for total and phosphorylated STAT3(Tyr705). GAPDH was used as endogenous control. A: representative immunoblots of each experimental sample; B: densitometric analysis (n=2).

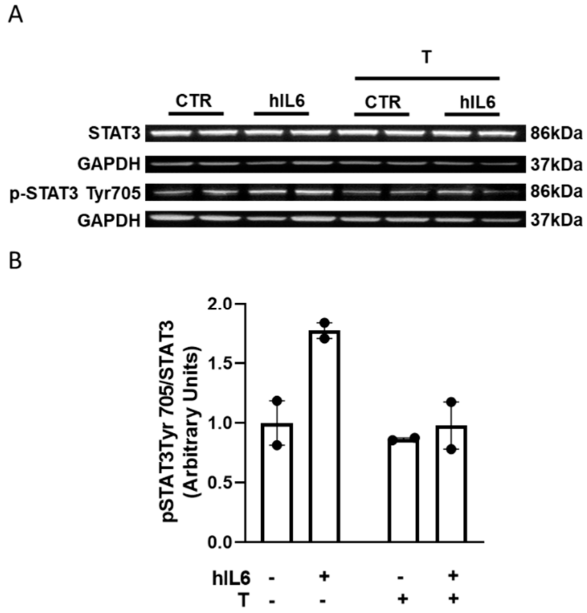

Table S1: Acute hIL6 decreases spontaneous AP frequency and amplitude of hiPSC-derived CMs and increases its duration. Detailed statistical analysis (Two-way ANOVA, Sidak's multiple comparisons

test) of acute hIL6 effect on AP frequency, amplitude, and duration at 90% of repolarization measured in hiPSC-derived CMs. CMs (differentiated for 39 days) were exposed to increasing hIL6 concentrations with or without 10 µg/ml Tocilizumab (T). AP durations at 90% of repolarization are corrected according to Bazett function. For AP frequency and duration n=10-13 and n=35-39, respectively, for hiPSC-CMs treated with hIL6 and hIL-6+T. ns: not significant.

	p value		
	Normalized AP frequency	Normalized AP amplitude	Corrected (Bazett) AP duration 90%
hIL6 0 pg/mL vs. T+hIL6 0 pg/mL	ns	ns	0,014
hIL6 0 pg/mL vs. hIL6 50 pg/mL	0,0023	<0,0001	ns
hIL6 0 pg/mL vs. T+hIL6 50 pg/mL	ns	ns	0,014
hIL6 0 pg/mL vs. hIL6 100 pg/mL	<0,0001	<0,0001	0,026
hIL6 0 pg/mL vs. T+hIL6 100 pg/mL	ns	0,002	0,002
hIL6 0 pg/mL vs. hIL6 500 pg/mL	<0,0001	<0,0001	0,001
hIL6 0 pg/mL vs. T+hIL6 500 pg/mL	0,038	<0,0001	<0,0001
hIL6 0 pg/mL vs. hIL6 1000 pg/mL	<0,0001	<0,0001	<0,0001
hIL6 0 pg/mL vs. T+hIL6 1000 pg/mL	<0,0001	<0,0001	<0,0001
hIL6 0 pg/mL vs. hIL6 10000 pg/mL	<0,0001	<0,0001	<0,0001
hIL6 0 pg/mL vs. T+hIL6 10000 pg/mL	<0,0001	<0,0001	<0,0001
hIL6 0 pg/mL vs. hIL6 50000 pg/mL	<0,0001	<0,0001	<0,0001
hIL6 0 pg/mL vs. T+hIL6 50000 pg/mL	<0,0001	<0,0001	<0,0001
hIL6 0 pg/mL vs. hIL6 100000 pg/mL	<0,0001	<0,0001	<0,0001
hIL6 0 pg/mL vs. T+hIL6 100000 pg/mL	<0,0001	<0,0001	<0,0001
hIL6 0 pg/mL vs. hIL6 500000 pg/mL	<0,0001	<0,0001	<0,0001
hIL6 0 pg/mL vs. T+hIL6 500000 pg/mL	<0,0001	<0,0001	<0,0001
T+hIL6 0 pg/mL vs. hIL6 50 pg/mL	0,011	<0,0001	ns
T+hIL6 0 pg/mL vs. T+hIL6 50 pg/mL	ns	ns	ns
T+hIL6 0 pg/mL vs. hIL6 100 pg/mL	<0,0001	<0,0001	ns

T+hIL6 0 pg/mL vs. T+hIL6 100 pg/mL	ns	0,011	ns
T+hIL6 0 pg/mL vs. hIL6 500 pg/mL	<0,0001	<0,0001	ns
T+hIL6 0 pg/mL vs. T+hIL6 500 pg/mL	ns	<0,0001	ns
T+hIL6 0 pg/mL vs. hIL6 1000 pg/mL	<0,0001	<0,0001	ns
T+hIL6 0 pg/mL vs. T+hIL6 1000 pg/mL	0,0001	<0,0001	ns
T+hIL6 0 pg/mL vs. hIL6 10000 pg/mL	<0,0001	<0,0001	<0,0001
T+hIL6 0 pg/mL vs. T+hIL6 10000 pg/mL	<0,0001	<0,0001	0,002
T+hIL6 0 pg/mL vs. hIL6 50000 pg/mL	<0,0001	<0,0001	<0,0001
T+hIL6 0 pg/mL vs. T+hIL6 50000 pg/mL	<0,0001	<0,0001	<0,0001
T+hIL6 0 pg/mL vs. hIL6 100000 pg/mL	<0,0001	<0,0001	<0,0001
T+hIL6 0 pg/mL vs. T+hIL6 100000 pg/mL	<0,0001	<0,0001	<0,0001
T+hIL6 0 pg/mL vs.hIL6 500000 pg/mL	<0,0001	<0,0001	<0,0001
T+hIL6 0 pg/mL vs. T+hIL6 500000 pg/mL	<0,0001	<0,0001	0,002
hIL6 50 pg/mL vs. T+hIL6 50 pg/mL	ns	<0,0001	ns
hIL6 50 pg/mL vs. hIL6 100 pg/mL	ns	ns	ns
hIL6 50 pg/mL vs. T+hIL6 100 pg/mL	ns	ns	ns
hIL6 50 pg/mL vs. hIL6 500 pg/mL	0,004	ns	ns
hIL6 50 pg/mL vs. T+hIL6 500 pg/mL	ns	ns	0,011
hIL6 50 pg/mL vs. hIL6 1000 pg/mL	<0,0001	ns	ns
hIL6 50 pg/mL vs. T+hIL6 1000 pg/mL	ns	ns	0,048
hIL6 50 pg/mL vs. hIL6 10000 pg/mL	<0,0001	0,0003	<0,0001
hIL6 50 pg/mL vs. T+hIL6 10000 pg/mL	ns	ns	<0,0001
hIL6 50 pg/mL vs. hIL6 50000 pg/mL	<0,0001	0,0016	<0,0001
hIL6 50 pg/mL vs. T+hIL6 50000 pg/mL	0,0052	0,0383	<0,0001
hIL6 50 pg/mL vs. hIL6 100000 pg/mL	<0,0001	<0,0001	<0,0001

hIL6 50 pg/mL vs. T+hIL6 100000 pg/mL	<0,0001	0,048	<0,0001
hIL6 50 pg/mL vs.hIL6 500000 pg/mL	<0,0001	0,0015	<0,0001
hIL6 50 pg/mL vs. T+hIL6 500000 pg/mL	<0,0001	0,0025	<0,0001
T+hIL6 50 pg/mL vs. hIL6 100 pg/mL	0,002	<0,0001	ns
T+hIL6 50 pg/mL vs. T+hIL6 100 pg/mL	ns	ns	ns
T+hIL6 50 pg/mL vs. hIL6 500 pg/mL	<0,0001	<0,0001	ns
T+hIL6 50 pg/mL vs. T+hIL6 500 pg/mL	ns	0,007	ns
T+hIL6 50 pg/mL vs. hIL6 1000 pg/mL	<0,0001	<0,0001	ns
T+hIL6 50 pg/mL vs. T+hIL6 1000 pg/mL	0,060	0,011	ns
T+hIL6 50 pg/mL vs. hIL6 10000 pg/mL	<0,0001	<0,0001	<0,0001
T+hIL6 50 pg/mL vs. T+hIL6 10000 pg/mL	<0,0001	<0,0001	0,002
T+hIL6 50 pg/mL vs. hIL6 50000 pg/mL	<0,0001	<0,0001	<0,0001
T+hIL6 50 pg/mL vs. T+hIL6 50000 pg/mL	<0,0001	<0,0001	<0,0001
T+hIL6 50 pg/mL vs. hIL6 100000 pg/mL	<0,0001	<0,0001	<0,0001
T+hIL6 50 pg/mL vs. T+hIL6 100000 pg/mL	<0,0001	<0,0001	<0,0001
T+hIL6 50 pg/mL vs. hIL6 500000 pg/mL	<0,0001	<0,0001	<0,0001
T+hIL6 50 pg/mL vs. T+hIL6 500000 pg/mL	<0,0001	<0,0001	0,002
hIL6 100 pg/mL vs. T+hIL6 100 pg/mL	ns	0,02	ns
hIL6 100 pg/mL vs. hIL6 500 pg/mL	ns	ns	ns
hIL6 100 pg/mL vs. T+hIL6 500 pg/mL	ns	ns	ns
hIL6 100 pg/mL vs. hIL6 1000 pg/mL	ns	ns	ns
hIL6 100 pg/mL vs. T+hIL6 1000 pg/mL	ns	ns	ns
hIL6 100 pg/mL vs. hIL6 10000 pg/mL	<0,0001	0,012	<0,0001
hIL6 100 pg/mL vs. T+hIL6 10000 pg/mL	ns	ns	0,0013
hIL6 100 pg/mL vs. hIL6 50000 pg/mL	<0,0001	0,033	<0,0001

hIL6 100 pg/mL vs. T+hIL6 50000 pg/mL	ns	ns	<0,0001
hIL6 100 pg/mL vs. hIL6 100000 pg/mL	<0,0001	0,002	<0,0001
hIL6 100 pg/mL vs. T+hIL6 100000 pg/mL	ns	ns	<0,0001
hIL6 100 pg/mL vs. hIL6 500000 pg/mL	<0,0001	0,014	<0,0001
hIL6 100 pg/mL vs. T+hIL6 500000 pg/mL	0,001	0,026	0,001
T+hIL6 100 pg/mL vs. hIL6 500 pg/mL	<0,0001	<0,0001	<0,0001
T+hIL6 100 pg/mL vs. T+hIL6 500 pg/mL	ns	ns	ns
T+hIL6 100 pg/mL vs. hIL6 1000 pg/mL	<0,0001	0,0002	ns
T+hIL6 100 pg/mL vs. T+hIL6 1000 pg/mL	ns	ns	ns
T+hIL6 100 pg/mL vs. hIL6 10000 pg/mL	<0,0001	<0,0001	<0,0001
T+hIL6 100 pg/mL vs. T+hIL6 10000 pg/mL	0,002	0,001	0,010
T+hIL6 100 pg/mL vs. hIL6 50000 pg/mL	<0,0001	<0,0001	<0,0001
T+hIL6 100 pg/mL vs. T+hIL6 50000 pg/mL	<0,0001	<0,0001	<0,0001
T+hIL6 100 pg/mL vs. hIL6 100000 pg/mL	<0,0001	<0,0001	<0,0001
T+hIL6 100 pg/mL vs. T+hIL6 100000 pg/mL	<0,0001	<0,0001	<0,0001
T+hIL6 100 pg/mL vs. hIL6 500000 pg/mL	<0,0001	<0,0001	<0,0001
T+hIL6 100 pg/mL vs. T+hIL6 500000 pg/mL	<0,0001	<0,0001	0,007
hIL6 500 pg/mL vs. T+hIL6 500 pg/mL	0,0002	ns	ns
hIL6 500 pg/mL vs. hIL61000 pg/mL	ns	ns	ns
hIL6 500 pg/mL vs. T+hIL6 1000 pg/mL	ns	ns	ns
hIL6 500 pg/mL vs. hIL6 10000 pg/mL	0,002	ns	<0,0001
hIL6 500 pg/mL vs. T+hIL6 10000 pg/mL	ns	ns	0,011
hIL6 500 pg/mL vs. hIL6 50000 pg/mL	<0,0001	ns	<0,0001
hIL6 500 pg/mL vs. T+hIL6 50000 pg/mL	ns	ns	<0,0001
hIL6 500 pg/mL vs. hIL6 100000 pg/mL	<0,0001	ns	<0,0001

hIL6 500 pg/mL vs. T+hIL6 100000 pg/mL	ns	ns	<0,0001
hIL6 500 pg/mL vs. hIL6 500000 pg/mL	<0,0001	ns	<0,0001
hIL6 500 pg/mL vs. T+hIL6 500000 pg/mL	ns	ns	0,008
T+hIL6 500 pg/mL vs. hIL6 1000 pg/mL	<0,0001	ns	ns
T+hIL6 500 pg/mL vs. T+hIL6 1000 pg/mL	ns	ns	ns
T+hIL6 500 pg/mL vs. hIL6 10000 pg/mL	<0,0001	<0,0001	0,0002
T+hIL6 500 pg/mL vs. T+hIL6 10000 pg/mL	0,0357	ns	ns
T+hIL6 500 pg/mL vs. hIL6 50000 pg/mL	<0,0001	<0,0001	<0,0001
T+hIL6 500 pg/mL vs. T+hIL6 50000 pg/mL	0,0003	0,001	0,0064
T+hIL6 500 pg/mL vs. hIL6 100000 pg/mL	<0,0001	<0,0001	<0,0001
T+hIL6 500 pg/mL vs. T+hIL6 100000 pg/mL	<0,0001	0,002	0,049
T+hIL6 500 pg/mL vs. hIL6 500000 pg/mL	<0,0001	<0,0001	<0,0001
T+hIL6 500 pg/mL vs. T+hIL6 500000 pg/mL	<0,0001	0,0001	ns
hIL6 1000 pg/mL vs. T+hIL6 1000 pg/mL	0,009	ns	ns
hIL6 1000 pg/mL vs. hIL6 10000 pg/mL	ns	ns	<0,0001
hIL6 1000 pg/mL vs. T+hIL6 10000 pg/mL	ns	ns	ns
hIL6 1000 pg/mL vs. hIL6 50000 pg/mL	0,006	ns	<0,0001
hIL6 1000 pg/mL vs. T+hIL6 50000 pg/mL	ns	ns	0,0003
hIL6 1000 pg/mL vs. hIL6 100000 pg/mL	0,0044	ns	<0,0001
hIL6 1000 pg/mL vs. T+hIL6 100000 pg/mL	ns	ns	0,004
hIL6 1000 pg/mL vs. hIL6 500000 pg/mL	<0,0001	ns	<0,0001
hIL6 1000 pg/mL vs. T+hIL6 500000 pg/mL	ns	ns	ns
T+hIL6 1000 pg/mL vs. hIL6 10000 pg/mL	<0,0001	<0,0001	<0,0001
T+hIL6 1000 pg/mL vs. T+hIL6 10000 pg/mL	ns	ns	ns
T+hIL6 1000 pg/mL vs. hIL6 50000 pg/mL	<0,0001	0,0004	<0,0001

T+hIL6 1000 pg/mL vs. T+hIL6 50000 pg/mL	ns	0,009	0,003
T+hIL6 1000 pg/mL vs. hIL6 100000 pg/mL	<0,0001	<0,0001	<0,0001
T+hIL6 1000 pg/mL vs. T+hIL6 100000 pg/mL	0,0001	0,011	0,023
T+hIL6 1000 pg/mL vs. hIL6. 500000 pg/mL	<0,0001	0,0004	<0,0001
T+hIL6 1000 pg/mL vs. T+hIL6 500000 pg/mL	<0,0001	0,001	ns
hIL6 10000 pg/mL vs. T+hIL6 10000 pg/mL	<0,0001	ns	ns
hIL6 10000 pg/mL vs. hIL6. 50000 pg/mL	ns	ns	ns
hIL6 10000 pg/mL vs. T+hIL6 50000 pg/mL	0,016	ns	ns
hIL6 10000 pg/mL vs. hIL6 100000 pg/mL	ns	ns	<0,0001
hIL6 10000 pg/mL vs. T+hIL6 100000 pg/mL	ns	ns	ns
hIL6 10000 pg/mL vs. hIL6 500000 pg/mL	ns	ns	0,0002
hIL6 10000 pg/mL vs. T+hIL6 500000 pg/mL	ns	ns	ns
T+hIL6 10000 pg/mL vs. hIL6 50000 pg/mL	<0,0001	ns	0,0001
T+hIL6 10000 pg/mL vs. T+hIL6 50000 pg/mL	ns	ns	ns
T+hIL6 10000 pg/mL vs. hIL6 100000 pg/mL	<0,0001	ns	<0,0001
T+hIL6 10000 pg/mL vs. T+hIL6 100000 pg/mL	ns	ns	ns
T+hIL6 10000 pg/mL vs hIL6. 500000 pg/mL	<0,0001	ns	<0,0001
T+hIL6 10000 pg/mL vs. T+hIL6 500000 pg/mL	0,003	ns	ns
hIL6 50000 pg/mL vs. T+hIL6 50000 pg/mL	0,001	ns	ns
hIL6 50000 pg/mL vs. hIL6 100000 pg/mL	ns	ns	ns
hIL6 50000 pg/mL vs. T+hIL6 100000 pg/mL	ns	ns	ns
hIL6 50000 pg/mL vs. hIL6 500000 pg/mL	ns	ns	ns
hIL6 50000 pg/mL vs. T+hIL6 500000 pg/mL	ns	ns	ns
T+hIL6 50000 pg/mL vs. hIL6 100000 pg/mL	0,0009	ns	<0,0001
T+hIL6 50000 pg/mL vs. T+hIL6 100000 pg/mL	ns	ns	ns

T+hIL6 50000 pg/mL vs. hIL6 500000 pg/mL	<0,0001	ns	<0,0001
T+hIL6 50000 pg/mL vs. T+hIL6 500000 pg/mL	ns	ns	ns
hIL6 100000 pg/mL vs. T+hIL6100000 pg/mL	ns	ns	<0,0001
hIL6 100000 pg/mL vs. hIL6 500000 pg/mL	ns	ns	ns
hIL6 100000 pg/mL vs. T+hIL6 500000 pg/mL	ns	ns	<0,0001
T+hIL6 100000 pg/mL vs. hIL6 500000 pg/mL	0,037	ns	<0,0001
T+hIL6 100000 pg/mL vs. T+hIL6 500000 pg/mL	ns	ns	ns
hIL6 500000 pg/mL vs. T+hIL6. 500000 pg/mL	ns	ns	0,0001

Figure S3: Acute hIL6 does not modify voltage dependence and kinetic properties of HCN4-mediated current. Potentials of half-maximal activation (V1/2, A) and activation constant (B) of HCN4-current recorded in control conditions and after exposure to hIL6 (from 0.05 to 50 ng/mL). Values are expressed as mean of 4-7 independent experiment ± standard error of the mean.

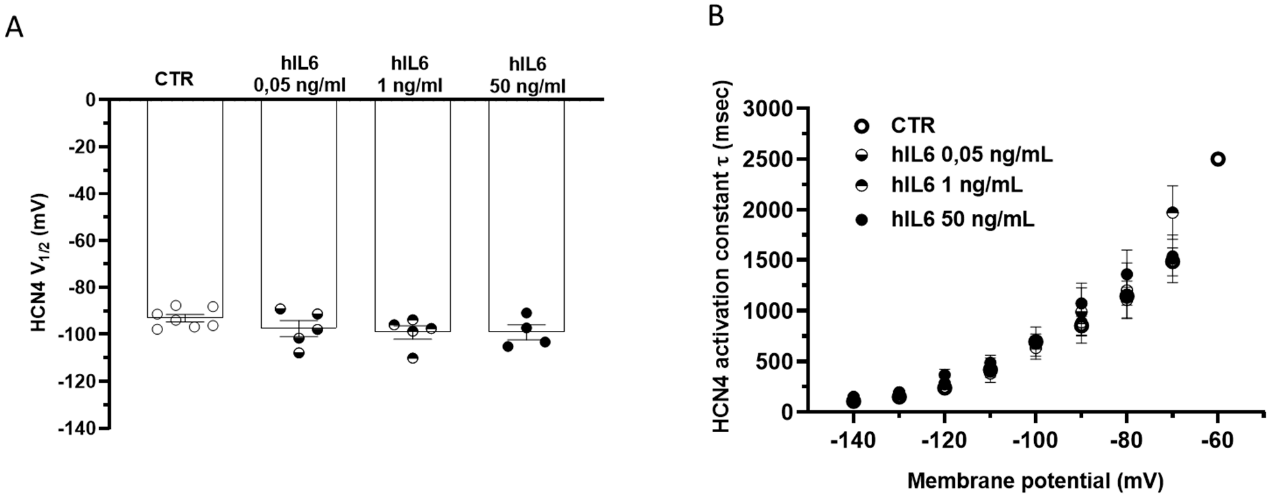

Table S2: Acute hIL6 decreases hHCN4-mediated current. Detailed statistical analysis (Two-way ANOVA, Sidak's multiple comparisons test) of acute hIL6 effect on hHCN4-mediated current recorded in HEK cells at different test potentials. ns: not significant.

	p value
-40 mV CTR vs. -40 mV hIL6 0,05 to 50 ng/mL	ns
-40 mV CTR vs.-50 mV CTR	ns
-40 mV CTR vs. -50 mV hIL6 0,05 to 50 ng/mL	ns
-40 mV CTR vs. -60 mV CTR	ns
-40 mV CTR vs. -60 mV hIL6 0,05 to 50 ng/mL	ns
-40 mV CTR vs. -70 mV CTR	ns
-40 mV CTR vs. -70 mV hIL6 0,05 to 50 ng/mL	ns
-40 mV CTR vs. -80 mV CTR	ns
-40 mV CTR vs. -80 mV hIL6 0,05 to 50 ng/mL	ns
-40 mV CTR vs. -90 mV CTR	<0,0001
-40 mV CTR vs. -90 mV hIL6 0,05 to 50 ng/mL	ns
-40 mV CTR vs. -100 mV CTR	<0,0001
-40 mV CTR vs. -100 mV hIL6 0,05 to 1 ng/mL	<0,0001
-40 mV CTR vs. -100 mV hIL6 50 ng/mL	0,0018
-40 mV CTR vs. -110 mV CTR	<0,0001
-40 mV CTR vs. -110 mV hIL6 0,05 to 50 ng/mL	<0,0001
-40 mV CTR vs. -120 mV CTR	<0,0001
-40 mV CTR vs. -120 mV hIL6 0,05 to 50 ng/mL	<0,0001
-40 mV CTR vs. -130 mV CTR	<0,0001
-40 mV CTR vs. -130 mV hIL6 0,05 to 50 ng/mL	<0,0001
-40 mV CTR vs. -140 mV CTR	<0,0001
-40 mV CTR vs. -140 mV hIL6 0,05 to 50 ng/mL	<0,0001
-40 mV hIL6 0,05 ng/mL vs. -40 mV hIL6 1 to 50 ng/mL	ns
-40 mV hIL6 0,05 ng/mL vs. -50 mV CTR	ns
-40 mV hIL6 0,05 ng/mL vs. -50 mV hIL6 0,05 to 50 ng/mL	ns

-40 mV hIL6 0,05 ng/mL vs. -60 mV CTR	ns
-40 mV hIL6 0,05 ng/mL vs. -60 mV hIL6 0,05 to 50 ng/mL	ns
-40 mV hIL6 0,05 ng/mL vs. -70 mV CTR	ns
-40 mV hIL6 0,05 ng/mL vs. -70 mV hIL6 0,05 to 50 ng/mL	ns
-40 mV hIL6 0,05 ng/mL vs. -80 mV CTR	ns
-40 mV hIL6 0,05 ng/mL vs. -80 mV hIL6 0,05 to 50 ng/mL	ns
-40 mV hIL6 0,05 ng/mL vs. -90 mV CTR	<0,0001
-40 mV hIL6 0,05 ng/mL vs. -90 mV hIL6 0,05 ng/mL	ns
-40 mV hIL6 0,05 ng/mL vs. -90 mV hIL6 1 ng/mL	ns
-40 mV hIL6 0,05 ng/mL vs. -90 mV hIL6 50 ng/mL	ns
-40 mV hIL6 0,05 ng/mL vs. -100 mV CTR	<0,0001
-40 mV hIL6 0,05 ng/mL vs. -100 mV hIL6 0,05 to 50 ng/mL	<0,0001
-40 mV hIL6 0,05 ng/mL vs. -110 mV CTR	<0,0001
-40 mV hIL6 0,05 ng/mL vs. -110 mV hIL6 0,05 to 50 ng/mL	<0,0001
-40 mV hIL6 0,05 ng/mL vs. -120 mV CTR	<0,0001
-40 mV hIL6 0,05 ng/mL vs. -120 mV hIL6 0,05 to 50 ng/mL	<0,0001
-40 mV hIL6 0,05 ng/mL vs. -130 mV CTR	<0,0001
-40 mV hIL6 0,05 ng/mL vs. -130 mV hIL6 0,05 to 50 ng/mL	<0,0001
-40 mV hIL6 0,05 ng/mL vs. -140 mV CTR	<0,0001
-40 mV hIL6 0,05 ng/mL vs. -140 mV hIL6 0,05 to 50 ng/mL	<0,0001
-40 mV hIL6 1 ng/mL vs. -40 mV hIL6 50 ng/mL	ns
-40 mV hIL6 1 ng/mL vs. -50 mV CTR	ns
-40 mV hIL6 1 ng/mL vs. -50 mV hIL6 0,05 to 50 ng/mL	ns
-40 mV hIL6 1 ng/mL vs. -60 mV CTR	ns
-40 mV hIL6 1 ng/mL vs. -60 mV hIL6 0,05 to 50 ng/mL	ns
-40 mV hIL6 1 ng/mL vs. -70 mV CTR	ns
-40 mV hIL6 1 ng/mL vs. -70 mV hIL6 0,05 to 50 ng/mL	ns
-40 mV hIL6 1 ng/mL vs. -70 mV hIL6 1 ng/mL	ns
-40 mV hIL6 1 ng/mL vs. -80 mV hIL6 0,05 to 50 ng/mL	ns

-40 mV hIL6 1 ng/mL vs. -90 mV CTR	<0,0001
-40 mV hIL6 1 ng/mL vs. -90 mV hIL6 0,05 to 50 ng/mL	ns
-40 mV hIL6 1 ng/mL vs. -100 mV CTR	<0,0001
-40 mV hIL6 1 ng/mL vs. -100 mV hIL6 0,05 to 50 ng/mL	<0,0001
-40 mV hIL6 1 ng/mL vs. -110 mV CTR	<0,0001
-40 mV hIL6 1 ng/mL vs. -110 mV hIL6 0,05 to 50 ng/mL	<0,0001
-40 mV hIL6 1 ng/mL vs. -120 mV CTR	<0,0001
-40 mV hIL6 1 ng/mL vs. -120 mV hIL6 0,05 to 50 ng/mL	<0,0001
-40 mV hIL6 1 ng/mL vs. -130 mV CTR	<0,0001
-40 mV hIL6 1 ng/mL vs. -130 mV hIL6 0,05 to 50 ng/mL	<0,0001
-40 mV hIL6 1 ng/mL vs. -140 mV CTR	<0,0001
-40 mV hIL6 1 ng/mL vs. -140 mV hIL6 0,05 to 50 ng/mL	<0,0001
-40 mV hIL6 50 ng/mL vs. -50 mV CTR	ns
-40 mV hIL6 50 ng/mL vs. -50 mV hIL6 0,05 to 50 ng/mL	ns
-40 mV hIL6 50 ng/mL vs. -60 mV CTR	ns
-40 mV hIL6 50 ng/mL vs. -60 mV hIL6 0,05 to 50 ng/mL	ns
-40 mV hIL6 50 ng/mL vs. -70 mV CTR	ns
-40 mV hIL6 50 ng/mL vs. -70 mV hIL6 0,05 to 50 ng/mL	ns
-40 mV hIL6 50 ng/mL vs. -80 mV CTR	ns
-40 mV hIL6 50 ng/mL vs. -80 mV hIL6 0,05 to 50 ng/mL	ns
-40 mV hIL6 50 ng/mL vs. -90 mV CTR	<0,0001
-40 mV hIL6 50 ng/mL vs. -90 mV hIL6 0,05 to 50 ng/mL	ns
-40 mV hIL6 50 ng/mL vs. -100 mV CTR	<0,0001
-40 mV hIL6 50 ng/mL vs. -100 mV hIL6 0,05 ng/mL	<0,0001
-40 mV hIL6 50 ng/mL vs. -100 mV hIL6 1 ng/mL	0,0003
-40 mV hIL6 50 ng/mL vs. -100 mV hIL6 50 ng/mL	0,016
-40 mV hIL6 50 ng/mL vs. -110 mV CTR	<0,0001
-40 mV hIL6 50 ng/mL vs. -110 mV hIL6 0,05 to 50 ng/mL	<0,0001
-40 mV hIL6 50 ng/mL vs. -120 mV CTR	<0,0001

-40 mV hIL6 50 ng/mL vs. -120 mV hIL6 0,05 to 50 ng/mL	<0,0001
-40 mV hIL6 50 ng/mL vs. -130 mV CTR	<0,0001
-40 mV hIL6 50 ng/mL vs. -130 mV hIL6 0,05 to 50 ng/mL	<0,0001
-40 mV hIL6 50 ng/mL vs. -140 mV CTR	<0,0001
-40 mV hIL6 50 ng/mL vs. -140 mV hIL6 0,05 to 50 ng/mL	<0,0001
-50 mV CTR vs. -50 mV hIL6 0,05 to 50 ng/mL	ns
-50 mV CTR vs. -60 mV CTR	ns
-50 mV CTR vs. -60 mV hIL6 0,05 to 50 ng/mL	ns
-50 mV CTR vs. -70 mV CTR	ns
-50 mV CTR vs. -70 mV hIL6 0,05 to 50 ng/mL	ns
-50 mV CTR vs. -80 mV CTR	ns
-50 mV CTR vs. -80 mV hIL6 0,05 to 50 ng/mL	ns
-50 mV CTR vs. -90 mV CTR	<0,0001
-50 mV CTR vs. -90 mV hIL6 0,05 to 50 ng/mL	ns
-50 mV CTR vs. -100 mV CTR	<0,0001
-50 mV CTR vs. -100 mV hIL6 0,05 to 50 ng/mL	<0,0001
-50 mV CTR vs. -110 mV CTR	<0,0001
-50 mV CTR vs. -110 mV hIL6 0,05 to 50 ng/mL	<0,0001
-50 mV CTR vs. -120 mV CTR	<0,0001
-50 mV CTR vs. -120 mV hIL6 0,05 to 50 ng/mL	<0,0001
-50 mV CTR vs. -130 mV CTR	<0,0001
-50 mV CTR vs. -130 mV hIL6 0,05 to 50 ng/mL	<0,0001
-50 mV CTR vs. -140 mV CTR	<0,0001
-50 mV CTR vs. -140 mV hIL6 0,05 to 50 ng/mL	<0,0001
-50 mV hIL6 0,05 ng/mL vs. -50 mV hIL6 1 to 50 ng/mL	ns
-50 mV hIL6 0,05 ng/mL vs. -60 mV CTR	ns
-50 mV hIL6 0,05 ng/mL vs. -60 mV hIL6 0,05 to 50 ng/mL	ns
-50 mV hIL6 0,05 ng/mL vs. -70 mV CTR	ns
-50 mV hIL6 0,05 ng/mL vs. -70 mV hIL6 0,05 to 50 ng/mL	ns

-50 mV hIL6 0,05 ng/mL vs. -80 mV CTR	ns
-50 mV hIL6 0,05 ng/mL vs. -80 mV hIL6 0,05 to 50 ng/mL	ns
-50 mV hIL6 0,05 ng/mL vs. -90 mV CTR	<0,0001
-50 mV hIL6 0,05 ng/mL vs. -90 mV hIL6 0,05 to 50 ng/mL	ns
-50 mV hIL6 0,05 ng/mL vs. -100 mV CTR	<0,0001
-50 mV hIL6 0,05 ng/mL vs. -100 mV hIL6 0,05 to 50 ng/mL	<0,0001
-50 mV hIL6 0,05 ng/mL vs. -110 mV CTR	<0,0001
-50 mV hIL6 0,05 ng/mL vs. -110 mV hIL6 0,05 to 50 ng/mL	<0,0001
-50 mV hIL6 0,05 ng/mL vs. -120 mV CTR	<0,0001
-50 mV hIL6 0,05 ng/mL vs. -120 mV hIL6 0,05 to 50 ng/mL	<0,0001
-50 mV hIL6 0,05 ng/mL vs. -130 mV CTR	<0,0001
-50 mV hIL6 0,05 ng/mL vs. -130 mV hIL6 0,05 to 50 ng/mL	<0,0001
-50 mV hIL6 0,05 ng/mL vs. -140 mV CTR	<0,0001
-50 mV hIL6 0,05 ng/mL vs. -140 mV hIL6 0,05 to 50 ng/mL	<0,0001
-50 mV hIL6 1 ng/mL vs. -50 mV hIL6 50 ng/mL	ns
-50 mV hIL6 1 ng/mL vs. -60 mV CTR	ns
-50 mV hIL6 1 ng/mL vs. -60 mV hIL6 0,05 to 50 ng/mL	ns
-50 mV hIL6 1 ng/mL vs. -70 mV CTR	ns
-50 mV hIL6 1 ng/mL vs. -70 mV hIL6 0,05 to 50 ng/mL	ns
-50 mV hIL6 1 ng/mL vs. -80 mV CTR	ns
-50 mV hIL6 1 ng/mL vs. -80 mV hIL6 0,05 to 50 ng/mL	ns
-50 mV hIL6 1 ng/mL vs. -90 mV CTR	<0,0001
-50 mV hIL6 1 ng/mL vs. -90 mV hIL6 0,05 to 50 ng/mL	ns
-50 mV hIL6 1 ng/mL vs. -100 mV CTR	<0,0001
-50 mV hIL6 1 ng/mL vs. -100 mV hIL6 0,05 to 50 ng/mL	<0,0001
-50 mV hIL6 1 ng/mL vs. -110 mV CTR	<0,0001
-50 mV hIL6 1 ng/mL vs. -110 mV hIL6 0,05 to 50 ng/mL	<0,0001
-50 mV hIL6 1 ng/mL vs. -120 mV CTR	<0,0001
-50 mV hIL6 1 ng/mL vs. -120 mV hIL6 0,05 to 50 ng/mL	<0,0001

-50 mV hIL6 1 ng/mL vs. -130 mV CTR	<0,0001
-50 mV hIL6 1 ng/mL vs. -130 mV hIL6 0,05 to 50 ng/mL	<0,0001
-50 mV hIL6 1 ng/mL vs. -140 mV CTR	<0,0001
-50 mV hIL6 1 ng/mL vs. -140 mV hIL6 0,05 to 50 ng/mL	<0,0001
-50 mV hIL6 50 ng/mL vs. -60 mV CTR	ns
-50 mV hIL6 50 ng/mL vs. -60 mV hIL6 0,05 to 50 ng/mL	ns
-50 mV hIL6 50 ng/mL vs. -70 mV CTR	ns
-50 mV hIL6 50 ng/mL vs. -70 mV hIL6 0,05 to 50 ng/mL	ns
-50 mV hIL6 50 ng/mL vs. -80 mV CTR	ns
-50 mV hIL6 50 ng/mL vs. -80 mV hIL6 0,05 to 50 ng/mL	ns
-50 mV hIL6 50 ng/mL vs. -90 mV CTR	<0,0001
-50 mV hIL6 50 ng/mL vs. -90 mV hIL6 0,05 to 50 ng/mL	ns
-50 mV hIL6 50 ng/mL vs. -100 mV CTR	<0,0001
-50 mV hIL6 50 ng/mL vs. -100 mV hIL6 0,05 ng/mL	<0,0001
-50 mV hIL6 50 ng/mL vs. -100 mV hIL6 1 ng/mL	0,0003
-50 mV hIL6 50 ng/mL vs. -100 mV hIL6 50 ng/mL	0,016
-50 mV hIL6 50 ng/mL vs. -110 mV CTR	<0,0001
-50 mV hIL6 50 ng/mL vs. -110 mV hIL6 0,05 to 50 ng/mL	<0,0001
-50 mV hIL6 50 ng/mL vs. -120 mV CTR	<0,0001
-50 mV hIL6 50 ng/mL vs. -120 mV hIL6 0,05 to 50 ng/mL	<0,0001
-50 mV hIL6 50 ng/mL vs. -130 mV CTR	<0,0001
-50 mV hIL6 50 ng/mL vs. -130 mV hIL6 0,05 to 50 ng/mL	<0,0001
-50 mV hIL6 50 ng/mL vs. -140 mV CTR	<0,0001
-50 mV hIL6 50 ng/mL vs. -140 mV hIL6 0,05 to 50 ng/mL	<0,0001
-60 mV CTR vs. -60 mV hIL6 0,05 to 50 ng/mL	ns
-60 mV CTR vs. -70 mV CTR	ns
-60 mV CTR vs. -70 mV hIL6 0,05 to 50 ng/mL	ns
-60 mV CTR vs. -80 mV CTR	ns
-60 mV CTR vs. -80 mV hIL6 0,05 to 50 ng/mL	ns

-60 mV CTR vs. -90 mV CTR	<0,0001
-60 mV CTR vs. -90 mV hIL6 0,05 to 50 ng/mL	ns
-60 mV CTR vs. -100 mV CTR	<0,0001
-60 mV CTR vs. -100 mV hIL6 0,05 to 1 ng/mL	<0,0001
-60 mV CTR vs. -100 mV hIL6 50 ng/mL	0,002
-60 mV CTR vs. -110 mV CTR	<0,0001
-60 mV CTR vs. -110 mV hIL6 0,05 to 50 ng/mL	<0,0001
-60 mV CTR vs. -120 mV CTR	<0,0001
-60 mV CTR vs. -120 mV hIL6 0,05 to 50 ng/mL	<0,0001
-60 mV CTR vs. -130 mV CTR	<0,0001
-60 mV CTR vs. -130 mV hIL6 0,05 to 50 ng/mL	<0,0001
-60 mV CTR vs. -140 mV CTR	<0,0001
-60 mV CTR vs. -140 mV hIL6 0,05 to 50 ng/mL	<0,0001
-60 mV hIL6 0,05 ng/mL vs. -60 mV hIL6 1 to 50 ng/mL	ns
-60 mV hIL6 0,05 ng/mL vs. -70 mV CTR	ns
-60 mV hIL6 0,05 ng/mL vs. -70 mV hIL6 0,05 to 50 ng/mL	ns
-60 mV hIL6 0,05 ng/mL vs. -80 mV CTR	ns
-60 mV hIL6 0,05 ng/mL vs. -80 mV hIL6 0,05 to 50 ng/mL	ns
-60 mV hIL6 0,05 ng/mL vs. -90 mV CTR	<0,0001
-60 mV hIL6 0,05 ng/mL vs. -90 mV hIL6 0,05 to 50 ng/mL	ns
-60 mV hIL6 0,05 ng/mL vs. -100 mV CTR	<0,0001
-60 mV hIL6 0,05 ng/mL vs. -100 mV hIL6 0,05 to 1 ng/mL	<0,0001
-60 mV hIL6 0,05 ng/mL vs. -100 mV hIL6 50 ng/mL	0,0066
-60 mV hIL6 0,05 ng/mL vs. -110 mV CTR	<0,0001
-60 mV hIL6 0,05 ng/mL vs. -110 mV hIL6 0,05 to 50 ng/mL	<0,0001
-60 mV hIL6 0,05 ng/mL vs. -120 mV CTR	<0,0001
-60 mV hIL6 0,05 ng/mL vs. -120 mV hIL6 0,05 to 50 ng/mL	<0,0001
-60 mV hIL6 0,05 ng/mL vs. -130 mV CTR	<0,0001
-60 mV hIL6 0,05 ng/mL vs. -130 mV hIL6 0,05 to 50 ng/mL	<0,0001

-60 mV hIL6 0,05 ng/mL vs. -140 mV CTR	<0,0001
-60 mV hIL6 0,05 ng/mL vs. -140 mV hIL6 0,05 to 50 ng/mL	<0,0001
-60 mV hIL6 1 ng/mL vs. -60 mV hIL6 50 ng/mL	ns
-60 mV hIL6 1 ng/mL vs. -70 mV CTR	ns
-60 mV hIL6 1 ng/mL vs. -70 mV hIL6 0,05 to 50 ng/mL	ns
-60 mV hIL6 1 ng/mL vs. -80 mV CTR	ns
-60 mV hIL6 1 ng/mL vs. -80 mV hIL6 0,05 to 50 ng/mL	ns
-60 mV hIL6 1 ng/mL vs. -90 mV CTR	<0,0001
-60 mV hIL6 1 ng/mL vs. -90 mV hIL6 0,05 to 50 ng/mL	ns
-60 mV hIL6 1 ng/mL vs. -100 mV CTR	<0,0001
-60 mV hIL6 1 ng/mL vs. -100 mV hIL6 0,05 to 1 ng/mL	<0,0001
-60 mV hIL6 1 ng/mL vs. -100 mV hIL6 50 ng/mL	0,0066
-60 mV hIL6 1 ng/mL vs. -110 mV CTR	<0,0001
-60 mV hIL6 1 ng/mL vs. -110 mV hIL6 0,05 to 50 ng/mL	<0,0001
-60 mV hIL6 1 ng/mL vs. -120 mV CTR	<0,0001
-60 mV hIL6 1 ng/mL vs. -120 mV hIL6 0,05 to 50 ng/mL	<0,0001
-60 mV hIL6 1 ng/mL vs. -130 mV CTR	<0,0001
-60 mV hIL6 1 ng/mL vs. -130 mV hIL6 0,05 to 50 ng/mL	<0,0001
-60 mV hIL6 1 ng/mL vs. -140 mV CTR	<0,0001
-60 mV hIL6 1 ng/mL vs. -140 mV hIL6 0,05 to 50 ng/mL	<0,0001
-60 mV hIL6 50 ng/mL vs. -70 mV CTR	ns
-60 mV hIL6 50 ng/mL vs. -70 mV hIL6 0,05 to 50 ng/mL	ns
-60 mV hIL6 50 ng/mL vs. -80 mV CTR	ns
-60 mV hIL6 50 ng/mL vs. -80 mV hIL6 0,05 to 50 ng/mL	ns
-60 mV hIL6 50 ng/mL vs. -90 mV CTR	<0,0001
-60 mV hIL6 50 ng/mL vs. -90 mV hIL6 0,05 to 50 ng/mL	ns
-60 mV hIL6 50 ng/mL vs. -100 mV CTR	<0,0001
-60 mV hIL6 50 ng/mL vs. -100 mV hIL6 0,05 ng/mL	<0,0001
-60 mV hIL6 50 ng/mL vs. -100 mV hIL6 1 ng/mL	0,0003

-60 mV hIL6 50 ng/mL vs. -100 mV hIL6 50 ng/mL	0,016
-60 mV hIL6 50 ng/mL vs. -110 mV CTR	<0,0001
-60 mV hIL6 50 ng/mL vs. -110 mV hIL6 0,05 to 50 ng/mL	<0,0001
-60 mV hIL6 50 ng/mL vs. -120 mV CTR	<0,0001
-60 mV hIL6 50 ng/mL vs. -120 mV hIL6 0,05 to 50 ng/mL	<0,0001
-60 mV hIL6 50 ng/mL vs. -130 mV CTR	<0,0001
-60 mV hIL6 50 ng/mL vs. -130 mV hIL6 0,05 to 50 ng/mL	<0,0001
-60 mV hIL6 50 ng/mL vs. -140 mV CTR	<0,0001
-60 mV hIL6 50 ng/mL vs. -140 mV hIL6 0,05 to 50 ng/mL	<0,0001
-70 mV CTR vs. -70 mV hIL6 0,05 to 50 ng/mL	ns
-70 mV CTR vs. -80 mV CTR	ns
-70 mV CTR vs. -80 mV hIL6 0,05 to 50 ng/mL	ns
-70 mV CTR vs. -90 mV CTR	<0,0001
-70 mV CTR vs. -90 mV hIL6 0,05 to 50 ng/mL	ns
-70 mV CTR vs. -100 mV CTR	<0,0001
-70 mV CTR vs. -100 mV hIL6 0,05 ng/mL	0,0001
-70 mV CTR vs. -100 mV hIL6 1 ng/mL	0,0007
-70 mV CTR vs. -100 mV hIL6 50 ng/mL	ns
-70 mV CTR vs. -110 mV CTR	<0,0001
-70 mV CTR vs. -110 mV hIL6 0,05 to 50 ng/mL	<0,0001
-70 mV CTR vs. -120 mV CTR	<0,0001
-70 mV CTR vs. -120 mV hIL6 0,05 to 50 ng/mL	<0,0001
-70 mV CTR vs. -130 mV CTR	<0,0001
-70 mV CTR vs. -130 mV hIL6 0,05 to 50 ng/mL	<0,0001
-70 mV CTR vs. -140 mV CTR	<0,0001
-70 mV CTR vs. -140 mV hIL6 0,05 to 50 ng/mL	<0,0001
-70 mV hIL6 0,05 ng/mL vs. -70 mV hIL6 1 to 50 ng/mL	ns
-70 mV hIL6 0,05 ng/mL vs. -80 mV CTR	ns
-70 mV hIL6 0,05 ng/mL vs. -80 mV hIL6 0,05 to 50 ng/mL	ns

-70 mV hIL6 0,05 ng/mL vs. -90 mV CTR	<0,0001
-70 mV hIL6 0,05 ng/mL vs. -90 mV hIL6 0,05 to 50 ng/mL	ns
-70 mV hIL6 0,05 ng/mL vs. -100 mV CTR	<0,0001
-70 mV hIL6 0,05 ng/mL vs. -100 mV hIL6 0,05 ng/mL	<0,0001
-70 mV hIL6 0,05 ng/mL vs. -100 mV hIL6 1 ng/mL	0,0001
-70 mV hIL6 0,05 ng/mL vs. -100 mV hIL6 50 ng/mL	0,0116
-70 mV hIL6 0,05 ng/mL vs. -110 mV CTR	<0,0001
-70 mV hIL6 0,05 ng/mL vs. -110 mV hIL6 0,05 to 50 ng/mL	<0,0001
-70 mV hIL6 0,05 ng/mL vs. -120 mV CTR	<0,0001
-70 mV hIL6 0,05 ng/mL vs. -120 mV hIL6 0,05 to 50 ng/mL	<0,0001
-70 mV hIL6 0,05 ng/mL vs. -130 mV CTR	<0,0001
-70 mV hIL6 0,05 ng/mL vs. -130 mV hIL6 0,05 to 50 ng/mL	<0,0001
-70 mV hIL6 0,05 ng/mL vs. -140 mV CTR	<0,0001
-70 mV hIL6 0,05 ng/mL vs. -140 mV hIL6 0,05 to 50 ng/mL	<0,0001
-70 mV hIL6 1 ng/mL vs. -70 mV hIL6 50 ng/mL	ns
-70 mV hIL6 1 ng/mL vs. -80 mV CTR	ns
-70 mV hIL6 1 ng/mL vs. -80 mV hIL6 0,05 to 50 ng/mL	ns
-70 mV hIL6 1 ng/mL vs. -90 mV CTR	<0,0001
-70 mV hIL6 1 ng/mL vs. -90 mV hIL6 0,05 to 50 ng/mL	ns
-70 mV hIL6 1 ng/mL vs. -100 mV CTR	<0,0001
-70 mV hIL6 1 ng/mL vs. -100 mV hIL6 0,05 to 1 ng/mL	<0,0001
-70 mV hIL6 1 ng/mL vs. -100 mV hIL6 50 ng/mL	0,012
-70 mV hIL6 1 ng/mL vs. -110 mV CTR	<0,0001
-70 mV hIL6 1 ng/mL vs. -110 mV hIL6 0,05 to 50 ng/mL	<0,0001
-70 mV hIL6 1 ng/mL vs. -120 mV CTR	<0,0001
-70 mV hIL6 1 ng/mL vs. -120 mV hIL6 0,05 to 50 ng/mL	<0,0001
-70 mV hIL6 1 ng/mL vs. -130 mV CTR	<0,0001
-70 mV hIL6 1 ng/mL vs. -130 mV hIL6 0,05 to 50 ng/mL	<0,0001
-70 mV hIL6 1 ng/mL vs. -140 mV CTR	<0,0001

-70 mV hIL6 1 ng/mL vs. -140 mV hIL6 0,05 to 50 ng/mL	<0,0001
-70 mV hIL6 50 ng/mL vs. -80 mV CTR	ns
-70 mV hIL6 50 ng/mL vs. -80 mV hIL6 0,05 to 50 ng/mL	ns
-70 mV hIL6 50 ng/mL vs. -90 mV CTR	<0,0001
-70 mV hIL6 50 ng/mL vs. -90 mV hIL6 0,05 to 50 ng/mL	ns
-70 mV hIL6 50 ng/mL vs. -100 mV CTR	<0,0001
-70 mV hIL6 50 ng/mL vs. -100 mV hIL6 0,05 ng/mL	0,0001
-70 mV hIL6 50 ng/mL vs. -100 mV hIL6 1 ng/mL	0,0006
-70 mV hIL6 50 ng/mL vs. -100 mV hIL6 50 ng/mL	0,0305
-70 mV hIL6 50 ng/mL vs. -110 mV CTR	<0,0001
-70 mV hIL6 50 ng/mL vs. -110 mV hIL6 0,05 to 50 ng/mL	<0,0001
-70 mV hIL6 50 ng/mL vs. -120 mV CTR	<0,0001
-70 mV hIL6 50 ng/mL vs. -120 mV hIL6 0,05 to 50 ng/mL	<0,0001
-70 mV hIL6 50 ng/mL vs. -130 mV CTR	<0,0001
-70 mV hIL6 50 ng/mL vs. -130 mV hIL6 0,05 to 50 ng/mL	<0,0001
-70 mV hIL6 50 ng/mL vs. -140 mV CTR	<0,0001
-70 mV hIL6 50 ng/mL vs. -140 mV hIL6 0,05 to 50 ng/mL	<0,0001
-80 mV CTR vs. -80 mV hIL6 0,05 to 50 ng/mL	ns
-80 mV CTR vs. -90 mV CTR	ns
-80 mV CTR vs. -90 mV hIL6 0,05 to 50 ng/mL	ns
-80 mV CTR vs. -100 mV CTR	<0,0001
-80 mV CTR vs. -100 mV hIL6 0,05 to 50 ng/mL	ns
-80 mV CTR vs. -110 mV CTR	<0,0001
-80 mV CTR vs. -110 mV hIL6 0,05 ng/mL	<0,0001
-80 mV CTR vs. -110 mV hIL6 1 ng/mL	0,0008
-80 mV CTR vs. -110 mV hIL6 50 ng/mL	ns
-80 mV CTR vs. -120 mV CTR	<0,0001
-80 mV CTR vs. -120 mV hIL6 0,05 to 1 ng/mL	<0,0001
-80 mV CTR vs. -120 mV hIL6 50 ng/mL	0,0156

-80 mV CTR vs. -130 mV CTR	<0,0001
-80 mV CTR vs. -130 mV hIL6 0,05 to 50 ng/mL	<0,0001
-80 mV CTR vs. -140 mV CTR	<0,0001
-80 mV CTR vs. -140 mV hIL6 0,05 to 1 ng/mL	<0,0001
-80 mV CTR vs. -140 mV hIL6 50 ng/mL	0,0162
-80 mV hIL6 0,05 ng/mL vs. -80 mV hIL6 1 to 50 ng/mL	ns
-80 mV hIL6 0,05 ng/mL vs. -90 mV CTR	0,0059
-80 mV hIL6 0,05 ng/mL vs. -90 mV hIL6 0,05 to 50 ng/mL	ns
-80 mV hIL6 0,05 ng/mL vs. -100 mV CTR	<0,0001
-80 mV hIL6 0,05 ng/mL vs. -100 mV hIL6 0,05 ng/mL	0,042
-80 mV hIL6 0,05 ng/mL vs. -100 mV hIL6 1 ng/mL	ns
-80 mV hIL6 0,05 ng/mL vs. -100 mV hIL6 50 ng/mL	ns
-80 mV hIL6 0,05 ng/mL vs. -110 mV CTR	<0,0001
-80 mV hIL6 0,05 ng/mL vs. -110 mV hIL6 0,05 to 1 ng/mL	<0,0001
-80 mV hIL6 0,05 ng/mL vs. -110 mV hIL6 50 ng/mL	ns
-80 mV hIL6 0,05 ng/mL vs. -120 mV CTR	<0,0001
-80 mV hIL6 0,05 ng/mL vs. -120 mV hIL6 0,05 to 50 ng/mL	<0,0001
-80 mV hIL6 0,05 ng/mL vs. -130 mV CTR	<0,0001
-80 mV hIL6 0,05 ng/mL vs. -130 mV hIL6 0,05 to 50 ng/mL	<0,0001
-80 mV hIL6 0,05 ng/mL vs. -140 mV CTR	<0,0001
-80 mV hIL6 0,05 ng/mL vs. -140 mV hIL6 0,05 to 50 ng/mL	<0,0001
-80 mV hIL6 1 ng/mL vs. -80 mV hIL6 50 ng/mL	ns
-80 mV hIL6 1 ng/mL vs. -90 mV CTR	0,0001
-80 mV hIL6 1 ng/mL vs. -90 mV hIL6 0,05 to 50 ng/mL	ns
-80 mV hIL6 1 ng/mL vs. -100 mV CTR	<0,0001
-80 mV hIL6 1 ng/mL vs. -100 mV hIL6 0,05 ng/mL	0,0016
-80 mV hIL6 1 ng/mL vs. -100 mV hIL6 1 ng/mL	0,0074
-80 mV hIL6 1 ng/mL vs. -100 mV hIL6 50 ng/mL	ns
-80 mV hIL6 1 ng/mL vs. -110 mV CTR	<0,0001

-80 mV hIL6 1 ng/mL vs. -110 mV hIL6 0,05 to 1 ng/mL	<0,0001
-80 mV hIL6 1 ng/mL vs. -110 mV hIL6 50 ng/mL	0,0035
-80 mV hIL6 1 ng/mL vs. -120 mV CTR	<0,0001
-80 mV hIL6 1 ng/mL vs. -120 mV hIL6 0,05 to 50 ng/mL	<0,0001
-80 mV hIL6 1 ng/mL vs. -130 mV CTR	<0,0001
-80 mV hIL6 1 ng/mL vs. -130 mV hIL6 0,05 to 50 ng/mL	<0,0001
-80 mV hIL6 1 ng/mL vs. -140 mV CTR	<0,0001
-80 mV hIL6 1 ng/mL vs. -140 mV hIL6 0,05 to 50 ng/mL	<0,0001
-80 mV hIL6 50 ng/mL vs. -90 mV CTR	<0,0001
-80 mV hIL6 50 ng/mL vs. -90 mV hIL6 0,05 to 50 ng/mL	ns
-80 mV hIL6 50 ng/mL vs. -100 mV CTR	<0,0001
-80 mV hIL6 50 ng/mL vs. -100 mV hIL6 0,05 ng/mL	0,0011
-80 mV hIL6 50 ng/mL vs. -100 mV hIL6 1 ng/mL	0,0048
-80 mV hIL6 50 ng/mL vs. -100 mV hIL6 50 ng/mL	ns
-80 mV hIL6 50 ng/mL vs. -110 mV CTR	<0,0001
-80 mV hIL6 50 ng/mL vs. -110 mV hIL6 0,05 to 1 ng/mL	<0,0001
-80 mV hIL6 50 ng/mL vs. -110 mV hIL6 50 ng/mL	0,0022
-80 mV hIL6 50 ng/mL vs. -120 mV CTR	<0,0001
-80 mV hIL6 50 ng/mL vs. -120 mV hIL6 0,05 to 50 ng/mL	<0,0001
-80 mV hIL6 50 ng/mL vs. -130 mV CTR	<0,0001
-80 mV hIL6 50 ng/mL vs. -130 mV hIL6 0,05 to 50 ng/mL	<0,0001
-80 mV hIL6 50 ng/mL vs. -140 mV CTR	<0,0001
-80 mV hIL6 50 ng/mL vs. -140 mV hIL6 0,05 to 50 ng/mL	<0,0001
-90 mV CTR vs. -90 mV hIL6 0,05 ng/mL	ns
-90 mV CTR vs. -90 mV hIL6 1 ng/mL	ns
-90 mV CTR vs. -90 mV hIL6 50 ng/mL	0,0222
-90 mV CTR vs. -100 mV CTR	ns
-90 mV CTR vs. -100 mV hIL6 0,05 to 50 ng/mL	ns
-90 mV CTR vs. -110 mV CTR	<0,0001

-90 mV CTR vs. -110 mV hIL6 0,05 to 50 ng/mL	ns
-90 mV CTR vs. -120 mV CTR	<0,0001
-90 mV CTR vs. -120 mV hIL6 0,05 to 50 ng/mL	ns
-90 mV CTR vs. -130 mV CTR	<0,0001
-90 mV CTR vs. -130 mV hIL6 0,05 ng/mL	0,0106
-90 mV CTR vs. -130 mV hIL6 1 to 50 ng/mL	ns
-90 mV CTR vs. -140 mV CTR	<0,0001
-90 mV CTR vs. -140 mV hIL6 0,05 ng/mL	0,0009
-90 mV CTR vs. -140 mV hIL6 1 to 50 ng/mL	ns
-90 mV hIL6 0,05 ng/mL vs. -90 mV hIL6 1 to 50 ng/mL	ns
-90 mV hIL6 0,05 ng/mL vs. -100 mV CTR	<0,0001
-90 mV hIL6 0,05 ng/mL vs. -100 mV hIL6 0,05 to 50 ng/mL	ns
-90 mV hIL6 0,05 ng/mL vs. -110 mV CTR	<0,0001
-90 mV hIL6 0,05 ng/mL vs. -110 mV hIL6 0,05 ng/mL	0,0003
-90 mV hIL6 0,05 ng/mL vs. -110 mV hIL6 1 ng/mL	0,0275
-90 mV hIL6 0,05 ng/mL vs. -110 mV hIL6 50 ng/mL	ns
-90 mV hIL6 0,05 ng/mL vs. -120 mV CTR	<0,0001
-90 mV hIL6 0,05 ng/mL vs. -120 mV hIL6 0,05 ng/mL	<0,0001
-90 mV hIL6 0,05 ng/mL vs. -120 mV hIL6 1 ng/mL	0,0002
-90 mV hIL6 0,05 ng/mL vs. -120 mV hIL6 50 ng/mL	ns
-90 mV hIL6 0,05 ng/mL vs. -130 mV CTR	<0,0001
-90 mV hIL6 0,05 ng/mL vs. -130 mV hIL6 0,05 to 1 ng/mL	<0,0001
-90 mV hIL6 0,05 ng/mL vs. -130 mV hIL6 50 ng/mL	ns
-90 mV hIL6 0,05 ng/mL vs. -140 mV CTR	<0,0001
-90 mV hIL6 0,05 ng/mL vs. -140 mV hIL6 0,05 to 1 ng/mL	<0,0001
-90 mV hIL6 0,05 ng/mL vs. -140 mV hIL6 50 ng/mL	ns
-90 mV hIL6 1 ng/mL vs. -90 mV hIL6 50 ng/mL	ns
-90 mV hIL6 1 ng/mL vs. -100 mV CTR	<0,0001
-90 mV hIL6 1 ng/mL vs. -100 mV hIL6 0,05 to 50 ng/mL	ns

-90 mV hIL6 1 ng/mL vs. -110 mV CTR	<0,0001
-90 mV hIL6 1 ng/mL vs. -110 mV hIL6 0,05 ng/mL	<0,0001
-90 mV hIL6 1 ng/mL vs. -110 mV hIL6 1 ng/mL	0,0048
-90 mV hIL6 1 ng/mL vs. -110 mV hIL6 50 ng/mL	ns
-90 mV hIL6 1 ng/mL vs. -120 mV CTR	<0,0001
-90 mV hIL6 1 ng/mL vs. -120 mV hIL6 0,05 to 1 ng/mL	<0,0001
-90 mV hIL6 1 ng/mL vs. -120 mV hIL6 50 ng/mL	0,0481
-90 mV hIL6 1 ng/mL vs. -130 mV CTR	<0,0001
-90 mV hIL6 1 ng/mL vs. -130 mV hIL6 0,05 to 1 ng/mL	<0,0001
-90 mV hIL6 1 ng/mL vs. -130 mV hIL6 50 ng/mL	0,0121
-90 mV hIL6 1 ng/mL vs. -140 mV CTR	<0,0001
-90 mV hIL6 1 ng/mL vs. -140 mV hIL6 0,05 to 1 ng/mL	<0,0001
-90 mV hIL6 1 ng/mL vs. -140 mV hIL6 50 ng/mL	0,0423
-90 mV hIL6 50 ng/mL vs. -100 mV CTR	<0,0001
-90 mV hIL6 50 ng/mL vs. -100 mV hIL6 0,05 to 50 ng/mL	ns
-90 mV hIL6 50 ng/mL vs. -110 mV CTR	<0,0001
-90 mV hIL6 50 ng/mL vs. -110 mV hIL6 0,05 to 1 ng/mL	<0,0001
-90 mV hIL6 50 ng/mL vs. -110 mV hIL6 50 ng/mL	ns
-90 mV hIL6 50 ng/mL vs. -120 mV CTR	<0,0001
-90 mV hIL6 50 ng/mL vs. -120 mV hIL6 0,05 to 50 ng/mL	<0,0001
-90 mV hIL6 50 ng/mL vs. -130 mV CTR	<0,0001
-90 mV hIL6 50 ng/mL vs. -130 mV hIL6 0,05 to 50 ng/mL	<0,0001
-90 mV hIL6 50 ng/mL vs. -140 mV CTR	<0,0001
-90 mV hIL6 50 ng/mL vs. -140 mV hIL6 0,05 to 50 ng/mL	<0,0001
-100 mV CTR vs. -100 mV hIL6 0,05 ng/mL	ns
-100 mV CTR vs. -100 mV hIL6 1 ng/mL	0,0295
-100 mV CTR vs. -100 mV hIL6 50 ng/mL	0,004
-100 mV CTR vs. -110 mV CTR	ns
-100 mV CTR vs. -110 mV hIL6 0,05 to 50 ng/mL	ns

-100 mV CTR vs. -120 mV CTR	0,0006
-100 mV CTR vs. -120 mV hIL6 0,05 to 50 ng/mL	ns
-100 mV CTR vs. -130 mV CTR	<0,0001
-100 mV CTR vs. -130 mV hIL6 0,05 to 50 ng/mL	ns
-100 mV CTR vs. -140 mV CTR	0,0001
-100 mV CTR vs. -140 mV hIL6 0,05 to 50 ng/mL	ns
-100 mV hIL6 0,05 ng/mL vs. -100 mV hIL6 1 to 50 ng/mL	ns
-100 mV hIL6 0,05 ng/mL vs. -110 mV CTR	<0,0001
-100 mV hIL6 0,05 ng/mL vs. -110 mV hIL6 0,05 to 50 ng/mL	ns
-100 mV hIL6 0,05 ng/mL vs. -120 mV CTR	<0,0001
-100 mV hIL6 0,05 ng/mL vs. -120 mV hIL6 0,05 to 50 ng/mL	ns
-100 mV hIL6 0,05 ng/mL vs. -130 mV CTR	<0,0001
-100 mV hIL6 0,05 ng/mL vs. -130 mV hIL6 0,05 ng/mL	0,0175
-100 mV hIL6 0,05 ng/mL vs. -130 mV hIL6 1 to 50 ng/mL	ns
-100 mV hIL6 0,05 ng/mL vs. -140 mV CTR	<0,0001
-100 mV hIL6 0,05 ng/mL vs. -140 mV hIL6 0,05 ng/mL	0,0019
-100 mV hIL6 0,05 ng/mL vs. -140 mV hIL6 1 to 50 ng/mL	ns
-100 mV hIL6 1 ng/mL vs. -100 mV hIL6 50 ng/mL	ns
-100 mV hIL6 1 ng/mL vs. -110 mV CTR	<0,0001
-100 mV hIL6 1 ng/mL vs. -110 mV hIL6 0,05 to 50 ng/mL	ns
-100 mV hIL6 1 ng/mL vs. -120 mV CTR	<0,0001
-100 mV hIL6 1 ng/mL vs. -120 mV hIL6 0,05 to 50 ng/mL	ns
-100 mV hIL6 1 ng/mL vs. -130 mV CTR	<0,0001
-100 mV hIL6 1 ng/mL vs. -130 mV hIL6 0,05 ng/mL	0,0042
-100 mV hIL6 1 ng/mL vs. -130 mV hIL6 1 ng/mL	0,0312
-100 mV hIL6 1 ng/mL vs. -130 mV hIL6 50 ng/mL	ns
-100 mV hIL6 1 ng/mL vs. -140 mV CTR	<0,0001
-100 mV hIL6 1 ng/mL vs. -140 mV hIL6 0,05 ng/mL	0,0004
-100 mV hIL6 1 ng/mL vs. -140 mV hIL6 1 ng/mL	0,0292

-100 mV hIL6 1 ng/mL vs. -140 mV hIL6 50 ng/mL	ns
-100 mV hIL6 50 ng/mL vs. -110 mV CTR	<0,0001
-100 mV hIL6 50 ng/mL vs. -110 mV hIL6 0,05 to 50 ng/mL	ns
-100 mV hIL6 50 ng/mL vs. -120 mV CTR	<0,0001
-100 mV hIL6 50 ng/mL vs. -120 mV hIL6 0,05 ng/mL	0,0124
-100 mV hIL6 50 ng/mL vs. -120 mV hIL6 1 to 50 ng/mL	ns
-100 mV hIL6 50 ng/mL vs. -130 mV CTR	<0,0001
-100 mV hIL6 50 ng/mL vs. -130 mV hIL6 0,05 ng/mL	0,0005
-100 mV hIL6 50 ng/mL vs. -130 mV hIL6 1 ng/mL	0,0046
-100 mV hIL6 50 ng/mL vs. -130 mV hIL6 50 ng/mL	ns
-100 mV hIL6 50 ng/mL vs. -140 mV CTR	<0,0001
-100 mV hIL6 50 ng/mL vs. -140 mV hIL6 0,05 ng/mL	<0,0001
-100 mV hIL6 50 ng/mL vs. -140 mV hIL6 1 ng/mL	0,0042
-100 mV hIL6 50 ng/mL vs. -140 mV hIL6 50 ng/mL	ns
-110 mV CTR vs. -110 mV hIL6 0,05 ng/mL	ns
-110 mV CTR vs. -110 mV hIL6 1 ng/mL	0,0089
-110 mV CTR vs. -110 mV hIL6 50 ng/mL	<0,0001
-110 mV CTR vs. -120 mV CTR	ns
-110 mV CTR vs. -120 mV hIL6 0,05 ng/mL	ns
-110 mV CTR vs. -120 mV hIL6 1 ng/mL	ns
-110 mV CTR vs. -120 mV hIL6 50 ng/mL	0,0062
-110 mV CTR vs. -130 mV CTR	ns
-110 mV CTR vs. -130 mV hIL6 0,05 to 1 ng/mL	ns
-110 mV CTR vs. -130 mV hIL6 50 ng/mL	0,0294
-110 mV CTR vs. -140 mV CTR	ns
-110 mV CTR vs. -140 mV hIL6 0,05 to 50 ng/mL	ns
-110 mV hIL6 0,05 ng/mL vs. -110 mV hIL6 1 to 50 ng/mL	ns
-110 mV hIL6 0,05 ng/mL vs. -120 mV CTR	0,0002
-110 mV hIL6 0,05 ng/mL vs. -120 mV hIL6 0,05 to 50 ng/mL	ns

-110 mV hIL6 0,05 ng/mL vs. -130 mV CTR	<0,0001
-110 mV hIL6 0,05 ng/mL vs. -130 mV hIL6 0,05 to 50 ng/mL	ns
-110 mV hIL6 0,05 ng/mL vs. -140 mV CTR	<0,0001
-110 mV hIL6 0,05 ng/mL vs. -140 mV hIL6 0,05 to 50 ng/mL	ns
-110 mV hIL6 1 ng/mL vs. -110 mV hIL6 50 ng/mL	ns
-110 mV hIL6 1 ng/mL vs. -120 mV CTR	<0,0001
-110 mV hIL6 1 ng/mL vs. -120 mV hIL6 0,05 to 50 ng/mL	ns
-110 mV hIL6 1 ng/mL vs. -130 mV CTR	<0,0001
-110 mV hIL6 1 ng/mL vs. -130 mV hIL6 0,05 to 50 ng/mL	ns
-110 mV hIL6 1 ng/mL vs. -140 mV CTR	<0,0001
-110 mV hIL6 1 ng/mL vs. -140 mV hIL6 0,05 to 50 ng/mL	ns
-110 mV hIL6 50 ng/mL vs. -120 mV CTR	<0,0001
-110 mV hIL6 50 ng/mL vs. -120 mV hIL6 0,05 to 50 ng/mL	ns
-110 mV hIL6 50 ng/mL vs. -130 mV CTR	<0,0001
-110 mV hIL6 50 ng/mL vs. -130 mV hIL6 0,05 to 50 ng/mL	ns
-110 mV hIL6 50 ng/mL vs. -140 mV CTR	<0,0001
-110 mV hIL6 50 ng/mL vs. -140 mV hIL6 0,05 ng/mL	0,0088
-110 mV hIL6 50 ng/mL vs. -140 mV hIL6 1 to 50 ng/mL	ns
-120 mV CTR vs. -120 mV hIL6 0,05 ng/mL	0,004
-120 mV CTR vs. -120 mV hIL6 1 ng/mL	0,0004
-120 mV CTR vs. -120 mV hIL6 50 ng/mL	<0,0001
-120 mV CTR vs. -130 mV CTR	ns
-120 mV CTR vs. -130 mV hIL6 0,05 ng/mL	ns
-120 mV CTR vs. -130 mV hIL6 1 ng/mL	0,013
-120 mV CTR vs. -130 mV hIL6 50 ng/mL	<0,0001
-120 mV CTR vs. -140 mV CTR	ns
-120 mV CTR vs. -140 mV hIL6 0,05 ng/mL	ns
-120 mV CTR vs. -140 mV hIL6 1 ng/mL	0,015
-120 mV CTR vs. -140 mV hIL6 50 ng/mL	0,0001

-120 mV hIL6 0,05 ng/mL vs. -120 mV hIL6 1 ng/mL	ns
-120 mV hIL6 0,05 ng/mL vs. -120 mV hIL6 50 ng/mL	ns
-120 mV hIL6 0,05 ng/mL vs. -130 mV CTR	0,001
-120 mV hIL6 0,05 ng/mL vs. -130 mV hIL6 0,05 to 50 ng/mL	ns
-120 mV hIL6 0,05 ng/mL vs. -140 mV CTR	0,001
-120 mV hIL6 0,05 ng/mL vs. -140 mV hIL6 0,05 to 50 ng/mL	ns
-120 mV hIL6 1 ng/mL vs. -120 mV hIL6 50 ng/mL	ns
-120 mV hIL6 1 ng/mL vs. -130 mV CTR	<0,0001
-120 mV hIL6 1 ng/mL vs. -130 mV hIL6 0,05 to 50 ng/mL	ns
-120 mV hIL6 1 ng/mL vs. -140 mV CTR	<0,0001
-120 mV hIL6 1 ng/mL vs. -140 mV hIL6 0,05 to 50 ng/mL	ns
-120 mV hIL6 50 ng/mL vs. -130 mV CTR	<0,0001
-120 mV hIL6 50 ng/mL vs. -130 mV hIL6 0,05 to 50 ng/mL	ns
-120 mV hIL6 50 ng/mL vs. -140 mV CTR	<0,0001
-120 mV hIL6 50 ng/mL vs. -140 mV hIL6 0,05 to 50 ng/mL	ns
-130 mV CTR vs. -130 mV hIL6 0,05 ng/mL	0,029
-130 mV CTR vs. -130 mV hIL6 1 ng/mL	0,003
-130 mV CTR vs. -130 mV hIL6 50 ng/mL	<0,0001
-130 mV CTR vs. -140 mV CTR	ns
-130 mV CTR vs. -140 mV hIL6 0,05 ng/mL	ns
-130 mV CTR vs. -140 mV hIL6 1 ng/mL	0,004
-130 mV CTR vs. -140 mV hIL6 50 ng/mL	<0,0001
-130 mV hIL6 0,05 ng/mL vs. -130 mV hIL6 1 to 50 ng/mL	ns
-130 mV hIL6 0,05 ng/mL vs. -140 mV CTR	0,033
-130 mV hIL6 0,05 ng/mL vs. -140 mV hIL6 0,05 to 50 ng/mL	ns
-130 mV hIL6 1 ng/mL vs. -130 mV hIL6 50 ng/mL	ns
-130 mV hIL6 1 ng/mL vs. -140 mV CTR	0,004
-130 mV hIL6 1 ng/mL vs. -140 mV hIL6 0,05 to 50 ng/mL	ns
-130 mV hIL6 50 ng/mL vs. -140 mV CTR	<0,0001

-130 mV hIL6 50 ng/mL vs. -140 mV hIL6 0,05 to 50 ng/mL	ns
-140 mV CTR vs. -140 mV hIL6 0,05 ng/mL	ns
-140 mV CTR vs. -140 mV hIL6 1 ng/mL	0,004
-140 mV CTR vs. -140 mV hIL6 50 ng/mL	<0,0001
-140 mV hIL6 0,05 ng/mL vs. -140 mV hIL6 1 to 50 ng/mL	ns

Table S3. Demographics Characteristics of MV patients included in the study.

Total number (m/f);<br>MV-l number (m/f)<br>MV-h number (m/f)	24 (18/6)<br>5 (5/0)<br>5 (3/2)
Age (min/max)<br>MV-l (min/max)<br>MV-h (min/max)	51/85<br>59/76<br>62/84
ECG (SR/pAF/cAF) before surgery<br>MV-l<br>MV-h	14/9/1<br>3/2<br>3/2
EF (min/max)<br>MV-l<br>MV-h	45/67<br>55/60<br>55/60
Medication (number of total, MV-l, MV-h patients)	
α-Adrenoreceptor antagonists	3, 0, 0
Antiaggregants	3, 0, 0
Anticoagulants	6, 0, 3
Antinflammatory steroid	2, 0, 1
Antiepileptic	2, 1, 0
Antidiabetics	1, 0, 0
ARBs/ACE-I/MRA	3/7/5; 0/3/1; 0/2/0
Aspirin	6, 1, 2
Beta blocker	10, 3, 3
Calcium channel blocker	4, 3, 0
Flecainide/Amiodarone	3/1; 1/0; 1/0
Digoxin	1, 0, 1
Diuretics	14, 3, 3

Levothyroxin	2, 0, 1
Proton pump inhibitors	9, 1, 3
Statins	5, 2, 3

MV: mitral valve replacement; MV-l: MV patient with low atrial IL6 expression; MV-h: MV patient with high atrial IL6 expression; EF: Ejection Fraction; ARBs: Type 1 angiotensin-II receptor antagonists; ACE-I: angiotensin converting enzyme inhibitors; MR: mineralocorticoid receptor antagonists.
